# Incidence of influenza virus infection among pregnant women: a systematic review

**DOI:** 10.1186/s12884-017-1333-5

**Published:** 2017-05-30

**Authors:** Mark A. Katz, Bradford D. Gessner, Jeanene Johnson, Becky Skidmore, Marian Knight, Niranjan Bhat, Helen Marshall, David J. Horne, Justin R. Ortiz, Deshayne B. Fell

**Affiliations:** 1 Department of Health Systems Management, Medical School for International Health, Ben Gurion University of the Negev, Beer Sheva, Israel; 2 University of Michigan School of Public Health, Ann Arbor, MI USA; 3 Agence de Médecine Préventive, Paris, France; 4 Independent consultant, Oakland, USA; 5 Independent consultant, Ottawa, Canada; 6 National Perinatal Epidemiology Unit (NPEU), University of Oxford, Oxford, United Kingdom; 7 Vaccine Access and Delivery Program, PATH, Seattle, WA USA; 8 Vaccinology and Immunology Research Trials Unit, Discipline of Paediatrics, Women’s and Children’s Hospital and University of Adelaide, Adelaide, Australia; 9 Robinson Research Institute, University of Adelaide, North Adelaide, Australia; 10 Department of Medicine, University of Washington, Seattle, USA; 11 Initiative for Vaccine Research, World Health Organization, Geneva, Switzerland; 12 Better Outcomes Registry & Network (BORN), CHEO Research Institute, Ottawa, Canada; 13 Department of Epidemiology, Biostatistics and Occupational Health, McGill University, Montreal, Canada; 14 Agence de Médecine Préventive, Anchorage, AK USA; 15 Los Gatos, CA USA; 16 Harborview Medical Center, University of Washington, Seattle, WA USA

## Abstract

**Background:**

The World Health Organization (WHO) considers pregnant women to be a risk group for severe influenza disease. We conducted a systematic review to evaluate influenza disease incidence in pregnant women in order to inform estimates of influenza vaccine impact for low-resource countries.

**Methods:**

We performed electronic literature searches, targeting studies on the following outcomes in pregnant women: attack rate, hospitalization rate, intensive care unit admission rate, mortality rate, and disability-adjusted life years lost. Only original studies published in peer-reviewed journals that had laboratory confirmation for influenza virus infection and included population-based incidence rates with denominator data were included. We summarized study characteristics in descriptive tables and outcome-specific Forest plots. We generated summary incidence rates using random effects models and assessed statistical heterogeneity by visual examination of Forest plots, and by *χ* ^2^ and I^2^ tests.

**Results:**

We identified 1543 articles, of which nine articles met the study inclusion criteria. Five were case series, three were cohort studies, and one was a randomized controlled trial. Eight studies were from high-income countries, and one was from an upper middle-income country. Six studies reported results for pandemic influenza, and three reported seasonal influenza. Statistical heterogeneity was high for all outcomes, and methodologies and duration of surveillance varied considerably among studies; therefore, we did not perform meta-analyses.

**Conclusions:**

Study quality was very low according to GRADE criteria. More data on influenza disease incidence in pregnant women, particularly in low- and middle-income countries and for seasonal influenza disease, are needed to inform public health decision-making.

**Electronic supplementary material:**

The online version of this article (doi:10.1186/s12884-017-1333-5) contains supplementary material, which is available to authorized users.

## Background

In 2012, the World Health Organization (WHO) updated its influenza vaccine position by recommending that countries considering the initiation or expansion of programmes for seasonal influenza vaccination should prioritize pregnant women over other high-risk groups [1]. This recommendation was based on evidence regarding influenza disease risk among pregnant women and their infants, influenza vaccine safety and effectiveness, and programmatic opportunities to reach this population in low- and middle-income countries.

In some countries, maternal influenza immunization has been recommended for a number of years [2, 3]. While the primary goal of these vaccine policies has been to protect pregnant women from severe outcomes related to influenza virus infection [4], many policy decisions to-date have been based on limited knowledge on the fundamental issue of incidence rates of influenza virus infection in pregnant women, including infections associated with hospital admission, intensive care unit (ICU) admission, death, and influenza-associated disability-adjusted life years (DALYs) lost. Laboratory confirmation of influenza in general, but particularly in pregnant women, has only recently been obtained as part of clinical studies. Various definitions of influenza infection have been used in the past, albeit with less certainty of diagnosis given lack of laboratory confirmation. Published studies, mainly from the 2009 influenza pandemic, have identified pregnant women as having high rates and risk of severe influenza virus infection [1]. Other studies have shown that influenza vaccination can prevent influenza illness in pregnant women [5, 6], and recent data show an additional benefit of influenza illness prevention in infants of immunized women up to 6 months of age [5, 7, 8]. Nevertheless, the anticipated impact that influenza vaccine programs would have on severe outcomes at a population level is unclear [9], and such data would be helpful to inform policy and investment decisions for maternal influenza immunization.

In 2014, WHO convened a working group to review the epidemiologic evidence for maternal influenza illness [10]. The objectives of the working group were to develop evidence-based assumptions for vaccine impact modelling studies of maternal influenza immunization programs, to evaluate the quality of existing data, and to identify data gaps. To inform this working group, we conducted a systematic review to assess the incidence of laboratory-confirmed influenza illness among pregnant women, categorized by disease severity.

## Methods

### Data sources and searches

We conducted a systematic review in accordance with the Preferred Reporting Items for Systematic Reviews and Meta-Analyses (PRISMA) reporting recommendations [11]. The search strategy was developed and tested through an iterative process by an experienced medical information specialist (BS) in consultation with the review team. We searched PubMed, Embase, the Cochrane Library, and CINAHL Plus with Full Text on February 20, 2015. A second, add-on search utilizing keywords for “disability-adjusted life years” (DALYs) was performed in the same databases on February 28, 2015. Searches were updated biweekly through September 2, 2015. Strategies utilized a combination of controlled vocabulary (e.g., “Influenza A Virus”, “Pregnancy Complications”, “Incidence”) and keywords (e.g., influenza, prenatal, rate). Vocabulary and syntax were adjusted across databases. No language or date restrictions were applied but animal-only studies and opinion pieces (comments, editorials, interviews) were removed from the search results where possible. We did not conduct a review of grey literature. Specific details regarding the strategies appear in Additional file 1.

Following de-duplication, search records were uploaded into Abstrackr [12], an online systematic review tool, and screened by two independent reviewers (MK, JJ) to identify studies for full-text review. The two independent reviewers (MK, JJ) each read all of the full-text manuscripts and performed the final selection of included manuscripts. Discussion between the two reviewers was used to resolve disagreements.

### Study selection

We targeted studies that, regardless of methodology, included original data on any of the following outcomes in pregnant women: influenza virus infection of any kind; symptomatic influenza virus infection; influenza virus infection associated with hospital admission; influenza virus infection associated with intensive care unit (ICU) admission; influenza-associated death; and influenza-associated disability-adjusted life years (DALYs) lost. Only studies that had laboratory confirmation for influenza virus infection and included population-based incidence rates with denominator data were included. We excluded editorials, commentaries, opinion pieces, narrative reviews, clinical practice guidelines, conference abstracts, or literature not in peer-reviewed journals.

### Data extraction and methodological quality assessment

A data collection form was developed to abstract information from each manuscript regarding study country; study population; hospitalization status; time period; subject inclusion and exclusion criteria; laboratory methods used to confirm influenza diagnosis; whether the study evaluated pandemic or seasonal influenza;[13] methodology for determining numerators and denominators for calculations; and crude and adjusted incidence rates, with 95% confidence intervals, for each outcome. For the purpose of this review, we considered 2009 pandemic influenza A H1N1 to be seasonal influenza after August 2010, when WHO declared the pandemic over [13].

Two independent reviewers (MK, JJ) evaluated the quality of each study. Cohort studies were assessed using the Newcastle-Ottawa Scale [14]. Randomized clinical trials were evaluated using the Cochrane Collaboration Risk of Bias Tool [15], and case series were assessed using the JBI Critical Appraisal Checklist for Descriptive/Case Series [16]. For all of these scales, lower scores imply higher risk of bias.

We also evaluated the quality of the evidence for each outcome using the Grading of Recommendations Assessment, Development and Evaluation (GRADE) assessment criteria – both the traditional criteria and the assessment criteria related to prognosis [17, 18]. GRADE was initially developed to provide methodological guidance in reviewing the quality of evidence for intervention research, and later adapted for use in evaluating diagnostic and prognostic studies [17]. The approach considers five factors that can influence our confidence in estimates of effects: (1) study design and limitations in study design, (2) inconsistency of results across studies, (3) indirectness of the evidence, (4) imprecision and (5) publication bias; and three factors related to observational studies: (1) large estimates of treatment, (2) a dose-response gradient and (3) plausible confounding that would increase confidence in an estimate [17]. In our review, beginning with cohort studies at a high level of evidence, according to the GRADE methodology, the level of confidence in the estimate of effects was downgraded according to the above criteria. Because we analysed influenza attack rates rather than effect estimates from interventions to prevent influenza, we could not apply the criteria relating to the magnitude and dose-response of the effect estimates. For each outcome, we applied a final GRADE assessment according to one of four categories-high quality, moderate quality, low quality or very low quality-based on the upgrading or downgrading of the quality of evidence for each outcome.

### Data synthesis and analysis

We summarized study characteristics in descriptive tables and outcome-specific forest plots. For each outcome of interest, we used incidence rates reported in the manuscript when they were available. When incidence rates were not provided, we computed incidence rates with 95% confidence intervals (CI) from raw data using the binomial exact method and the variance stabilizing transformation for proportions, as proposed by Freeman and Tukey,[19] using the Stata user command “metaprop” [20]. We generated summary incidence rates using random effects models and assessed statistical heterogeneity by visual examination of forest plots, and by *χ* ^2^ and I^2^ tests [21, 22]. We did not pool estimates if methodological or statistical heterogeneity was high. We considered statistical heterogeneity to be high if the I^2^ value was ≥75%[23]. Forest plots were prepared using the R package “ggplot2.”

## Results

### Overall results

**Fig. 1:**
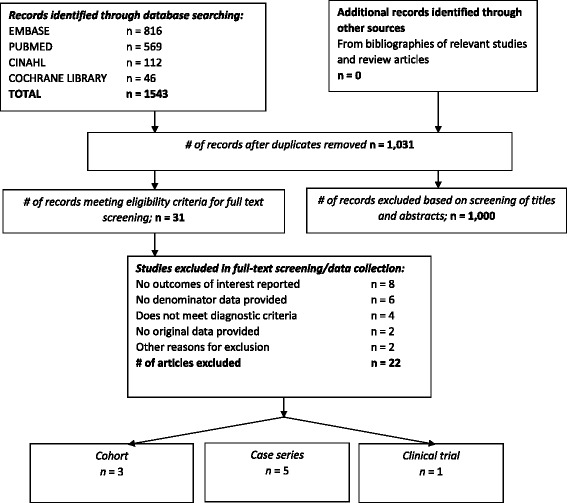
Flow diagram of systematic literature review of influenza illness in pregnancy

Our search results yielded 1543 records. After excluding duplicates, we screened 1041 titles and abstracts. We identified 31 potentially relevant articles, and, after reviewing the full manuscripts, we found 10 articles that met the study inclusion criteria (Fig. 1). The results of one study from Australia and New Zealand [24] were subsequently found to be included in another comparative study, [25] so the former study was excluded, leaving nine total articles. Three were cohort studies, [28–30] five were case series, [26, 27] and one was a randomized controlled trial [5].

Four studies were from the United States,[26, 29, 31, 32] three were from the United Kingdom,[27, 28, 30] one was from South Africa,[5] and one included data from Australia, New Zealand and the United Kingdom [25]. All studies were from high-income countries, except for the one study from South Africa, an upper-middle income country [33]. Six studies included pandemic influenza data, [25–27, 29–32] and three studies included seasonal influenza data, [5, 28, 30] [5]. Among the nine studies, three studies reported rates of influenza virus infection, including asymptomatic infection;[28–30] three studies reported rates of symptomatic influenza virus infection;[5, 26, 32] four studies reported influenza-associated hospital admission rates;[26, 27, 31, 32] four studies reported influenza-associated ICU admission rates;[25, 26, 31, 32] and four studies reported influenza-associated mortality rates [25, 26, 31, 32]. We did not identify any relevant studies that reported DALYs associated with influenza virus infection.

The methodologic approach to calculating denominator data varied among the studies (Table 1). Only one study defined the specific testing that was required for the determination of pregnancy [5].

**Table 1:** Summary of studies identified in systematic literature review of Incidence of influenza virus infection among pregnant women

Study	Outcome assessed^a^	Pandemic or seasonal	Study Design	Methodology	Denominator calculation	Dates of surveillance	Lab Test	Country (WHO Region) ^b^	Country Income Level
Creanga 2010	HA, ICU, D	Pandemic	Case series	Retrospective chart review: pregnant women hospitalized with confirmed H1N1 identified through “enhanced surveillance.” Testing performance only when requested by hospital.	Estimation using 2007 vital registration data (similar approach as in Jamieson et al.)	05/01/2009–06/30/2009	RT-PCR	US (PAHO)	High
Doyle 2013	SI, HA, ICU, D	Pandemic	Case series	Cases of pH1N1-confirmed acute respiratory illness reported by health providers to state of Florida	Florida birth registry data	04/24/2009–05/31/2010	RT-PCR or viral culture	US (PAHO)	High
Griffiths 1980	I	Seasonal	Cohort	Serological testing of women “booked” for antenatal care at one hospital in England during three years. Only women with postpartum specimens available were included.	Number of study participants	05/1975–11/1975; 12/1975–06/1976; 08/1976–04/1977	HI serology with 4-fold rise in antibody titer	UK (EURO)	High
Hardy 1961	I	Pandemic	Cohort	All pregnant women treated at Johns Hopkins Obstetrical Prenatal Clinic, with blood samples taken and questionnaires about symptoms administered monthly through delivery. Anyone with high HI titer was considered a case.	Number of study participants	10/14/1957–02/01/1958	Complement Complement fixation or HI test; no increase in titers required to be considered a case	US (PAHO)	High
Irving 2000	I	Seasonal	Cohort	Women delivering at two hospitals in Nottingham, England with available antenatal and postnatal sera.	Number of study participants	05/1993–07/1994	Single radial hemolysis and paired specimens for HI titer	UK (EURO)	High
Jamieson 2009	SI, HA, ICU, D	Pandemic	Case series	Pregnant women with confirmed or probable H1 infection reported by states to CDC as part of “enhanced” surveillance.	US Census bureau data	04/15/2009–05/18/2009	Confirmed: RT-PCR or viral culture+for H1. Probable: RT-PCR flu A+ but (−) for known subtypes	US (PAHO)	High
Knight 2011	ICU, D	Pandemic	Case series	Cases of H1 lab-confirmed ICU admission among pregnant women in Australia and New Zealand reported to Australian and New Zealand Intensive Care (ANZIC) study. Cases in UK reported to UK Obstetric Surveillance System.	National statistics on pregnancies combined with total birth data	AUS/NZ: 06/01/2009–08/31/2009 UK: 09/01/2009–01/31/2010	Not stated in manuscript (but referenced)	Australia and New Zealand (WPRO) UK (EURO)	High
Madhi 2014	SI	Seasonal	Randomized Clinical trial	RCT of influenza vaccine efficacy. All participants with ILI or unknown respiratory illness were tested for influenza.	All women enrolled in placebo arm of study	03/2011–08/2011; 03/2012–07/2012	RT-PCR	South Africa (AFRO)	Upper-Middle
Yates 2010^c^	HA, ICU, D	Pandemic	Case series	Clinician-reported of H1-confirmed pregnant women admitted to 221/223 hospitals with consultant-led maternity units in the UK. Surveillance included zero-reporting and follow-up of reported cases.	Birth data from UK office for national statistics	09/01/2009–01/31/2010	Not specified	UK (EURO)	High

^a^Outcomes are abbreviated as follows: *I* Infection, *SI* Symptomatic Infection, *HA* Hospital admission, *ICU* ICU admission, *D* Death

^b^WHO regions are abbreviated as follows: *PAHO* Americas, *EURO* Europe, *AFRO* Africa, *WPRO* Western Pacific

^c^The Yates study described same population as described in Knight study; therefore only Knight study results were included in the ICU and death tables

Applying the GRADE framework, all outcomes were deemed to be based on a very low quality of evidence (Additional file 2: Table S1). We based this determination on a number of factors, which collectively determined the final assessment: in most of the studies, there were significant risks of diagnostic ascertainment bias related to the frequency of testing patients and the variability of reporting results. In addition, the reporting period varied in many of the studies evaluating the same outcome, making it difficult to compare the same outcome measure across studies. There was high heterogeneity in results across studies measuring the same outcome. Finally, many of the studies were case studies, and the sample size in a number of the studies was quite small (see study sample sizes in Additional file 2).

The Newcastle Ottawa scores for the five observational studies ranged from 6 to 9, out of a maximum of 9. The observational studies had suboptimal representativeness of study participants [28–30], inadequate assessment of outcome [29], or important diagnostic ascertainment biases [26, 27]. Each of the three case series had a score of 6 out of 7 according to the JBI Critical Appraisal Checklist [16]. All but one [26] of the studies were carried out over relatively short time periods that captured only a portion of the period of activity of seasonal or pandemic influenza. For the five outcomes of interest that the studies addressed, the I^2^ test demonstrated high statistical heterogeneity, ranging from 93.1% (mortality) to 99.7% (hospitalization) (Additional file 2). Because of this high heterogeneity for each outcome, and because most studies within each outcome employed different methodologies-different approaches to diagnostic ascertainment and different durations of follow-up-we did not pool incidence rates for any of the outcomes.

### Incidence of influenza virus infection

**Fig. 2:**
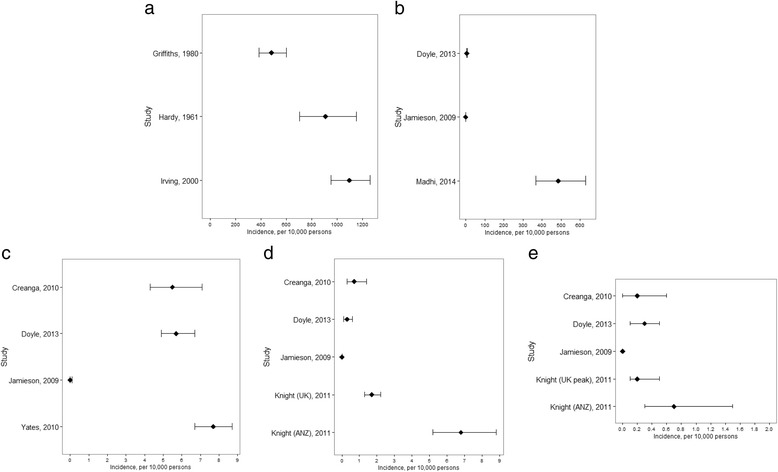
**a** Forest plot showing distribution of incidence of influenza virus infectionof any kind from serology studies in pregnant women, per 10,000 pregnancies, with 95% confidence intervals. **b** Forest plot showing distribution of incidence of symptomatic influenza virus infections in pregnant women, per 10,000 pregnancies, with 95% confidence intervals. **c** Forest plot showing distribution of incidence of influenza-associated hospital admissions in pregnant women, per 10,000 pregnancies, with 95% confidence intervals. **d** Forest plot showing distribution of incidence of influenza-associated ICU admissions in pregnant women, per 10,000 pregnancies, with 95% confidence intervals. **e** Forest plot showing distribution of incidence of influenza-associated deaths in pregnant women, per 10,000 pregnancies, with 95% confidence intervals

We identified three articles that described rates of serologically confirmed influenza virus infection, including clinical and asymptomatic infection, in pregnant women (Table 2, Fig. 2a) [28, 30]. Two studies reported rates for serologically confirmed seasonal influenza [28, 30]. and the Hardy et al. study described serologically confirmed influenza data from the 1957 influenza pandemic [29]. The two studies reporting on seasonal influenza virus infection did not mention the women’s vaccination status [28, 30], while the pandemic study excluded the small number of vaccinated women from the analysis [29]. These three studies used serological testing to confirm influenza virus infection, while the other studies use RT-PCR to confirm influenza virus infection. In all three serological studies, influenza virus infection incidence ranged from 483 to 1,097 cases per 10,000 pregnancies. The three serological studies had Newcastle Ottawa scores of 6 to 7. Across studies both the testing methodology and the sampling frame varied (Additional file 2, Table S1).

**Table 2:** Summary of studies reporting reporting incidence of influenza virus infection using serology

Study	Number of cases	Denominator	Crude incidence rate (CI) (^a^or derived from %)	95% CI	Pandemic or seasonal?	Study design	Grading system and score^b^	Hospital-basSummary of studies reporting repored or clinic-based?	Duration of surveillance (months or years)	Country
Griffiths 1980	77	1,595	483 per 10,000 pregnancies^a^	(399–614)	Seasonal	Cohort	NOS = 7	Hospital	3 years (22 months total influenza season)	UK
Hardy 1961	61	671	909 per 10,000 pregnancies^a^	(716–1,155)	Pandemic	Cohort	NOS = 6	Clinic	5 months	US
Irving 2000	182	1,659	1,097 per 10,000 pregnancies^a^	(957–1,258)	Seasonal	Cohort	NOS = 7	Hospital	15 months	UK

^a^Calculated from provided number of cases or %

^b^ *NOS* Newcastle-Ottawa Scale

### Incidence of symptomatic influenza virus infection

Three studies-two observational studies describing 2009 pandemic influenza (Doyle, 2013, and Jamieson, 2009) [26, 32] and one randomized clinical trial-Madhi et al. [5]—that described seasonal influenza-reported rates for symptomatic influenza virus infection among pregnant women in South Africa (Table 3, Fig. 2b). The two observational studies together reported a total of 128 confirmed cases of symptomatic influenza virus infection, and had a combined denominator of nearly 3.7 million pregnant women. In contrast, the clinical trial from South Africa included a total of 2,310 HIV-infected and uninfected pregnant women and reported 80 cases of symptomatic influenza virus infection among vaccinated and unvaccinated women combined. The two observational studies [26, 32] reported rates of 0.10 and 6.6 cases of symptomatic influenza virus infection per 10,000 pregnancies. The trial from South Africa [5] found an annual incidence of 371 cases of symptomatic influenza virus infection per 10,000 pregnancies among HIV-uninfected women and 1,818 cases of symptomatic influenza virus infection per 10,000 pregnancies among HIV-infected pregnant women. Approaches to case ascertainment varied considerably among the three studies; in the two observational studies (Doyle, 2013, and Jamieson, 2009), providers had to initiate testing for 2009 pandemic (H1N1) influenza through local or state public health laboratories, which then reported the results to the state health department or the US Centers for Disease Control. In contrast, during the South African vaccine trial, all participants were questioned weekly by study staff about symptoms consistent with influenza-like illness (ILI), and patients with ILI were immediately sampled for influenza testing.

**Table 3:** Summary of studies reporting incidence of symptomatic influenza virus infection

Study	Number of cases	Denominator	Crude incidence rate (CI)	95% CI	Pandemic or seasonal?	Study design	Grading system and score^b^	Hospital-based or clinic-based?	Duration of surveillance (months or years)	Country
Doyle 2013	194	295,941	6.6 per 10,000 pregnancies^a^	(5.70–7.55)	Pandemic	Case series	NOS = 8	Both	13 months	US
Jamieson 2009	34	3,392,060	0.10 per 10,000 pregnancies	(0.07–0.14)	Pandemic	Case series	JBI = 6/7	Both	1 month	US
Madhi 2014	HIV uninfected: 38	1,023	371 per 10,000 pregnancies^a^	(272–507)	Seasonal	Clinical trial	Low Risk (Cochrane)	Clinic	2 years (9 months total influenza season)	South Africa
	HIV infected: 16	88	1,818 per 10,000 pregnancies^a^	(1,167–2,832)						
	Total: 54	1,111	486 per 10,000 pregnancies^a^	(375–630)						

^a^Calculated from provided number of cases or %

^b^ *NOS* Newcastle-Ottawa Scale, *JBI* JBI Critical Appraisal Checklist for Descriptive/Case Series, *Cochrane* The Cochrane Collaboration’s tool for assessing risk of bias in randomised trials

### Incidence of influenza-associated hospital admissions

We identified four studies-all case series (Jamieson, 2009, Creanga, 2010, Yates, 2010, and Doyle, 2013)-that described influenza-associated hospital admission rates (Table 4, Fig. 2c) [26, 27, 31, 32]. All four included data from the 2009 influenza pandemic. Incidence rates of influenza-associated hospitalizations ranged from 0.04 to 7.7 cases per 10,000 pregnancies. While all four reported rates for 2009 pandemic (H1N1) influenza, the duration of surveillance varied among the studies, from one month [32] to 13 months [26]. In addition, methods of case ascertainment varied among the four studies. The three studies from the United States relied on passive reporting during the influenza pandemic, all to different government health authorities-local [31] state [26], and federal [32]—and all covered slightly different periods of the pandemic. As opposed to the United States studies, the Yates et al. study from the United Kingdom reflected the second wave of the pandemic [27]. The overall number of cases of influenza-associated hospital admission ranged from 14 to 271 in the four studies. Across studies, there was a high risk of diagnostic ascertainment bias related to the inconsistent frequency of testing patients and variability of reporting results to city, state, and federal public health authorities (Additional file 2, Table S3).

**Table 4:** Summary of studies reporting incidence of influenza-associated hospital admission

Study	Number of cases	Denominator	Crude incidence rate (CI)	95% CI	Pandemic or seasonal?	Study design	Grading system and score^b^	Hospital-based or clinic-based?	Duration of surveillance (months or years)	Country
Jamieson 2009	14	3,392,060	0.04 per 10,000 pregnancies	(0.02–0.07)	Pandemic	Case series	JBI = 6/7	Both	1 month	US
Creanga 2010	63	113,877	5.5 per 10,000 pregnancies	(4.25–7.08)	Pandemic	Case series	JBI = 6/7	Hospital	2 months	US
Yates 2010	241	314,135	7.7 per 10,000 pregnancies	(6.7–8.7)	Pandemic	Case series	NOS = 9	Hospital	5 months	UK
Doyle 2013	170	295,941	5.7 per 10,000 pregnancies^a^	(4.94–6.68)	Pandemic	Case series	NOS = 8	Both	13 months	US

^a^Calculated from provided number of cases or %

^b^ *NOS* Newcastle-Ottawa Scale, *JBI* JBI Critical Appraisal Checklist for Descriptive/Case Series

### Incidence of influenza-associated ICU admissions

Four studies (Jamieson, 2009, Creanga, 2010, Doyle, 2013, and Knight, 2011) described influenza-associated ICU admissions among pregnant women (Table 5, Fig. 2d) [25, 26, 31, 32]. The four studies, all case series, were all conducted during the 2009 influenza pandemic and reported rates of 0.01 to 6.8 cases of influenza-associated ICU admissions per 10,000 pregnancies. The same four studies also reported rates of influenza mortality among pregnant women during the 2009 influenza pandemic, which ranged from 0.03 to 0.69 influenza-associated deaths per 10,000 pregnancies (Table 6, Fig. 2e). Methodologies for case detection and the duration of surveillance varied among the studies. Two studies from the United States reported findings from the initial month [32] or two months [31] of the pandemic, while Knight et al. reported rates from the United Kingdom during a surveillance period that occurred during the second, smaller peak of the pandemic [25]. The absolute number of laboratory-confirmed influenza ICU admissions and laboratory-confirmed influenza deaths was small; the total number of influenza-associated ICU cases in each of the four studies ranged from 3 to 59, while the total number of influenza-associated deaths in each of the studies ranged from 1 to 6. (Additional file 2: Tables S4 and S5).

**Table 5:** Summary of studies reporting incidence of influenza-associated ICU admission

Study	Number of cases	Denominator	Crude incidence rate (CI)	95% CI	Pandemic or seasonal?	Study design	Grading system and score^b^	Hospital-based or clinic-based?	Duration of surveillance (months or years)	Country
Jamieson 2009	3	3,392,060	0.01 per 10,000 pregnancies^a^	(0.00–0.03)	Pandemic	Case series	JBI = 6/7	Both	1 month	US
Creanga 2010	8	113,877	0.70 per 10,000 pregnancies	(0.30–1.38)	Pandemic	Case series	JBI = 6/7	Hospital	2 months	US
Doyle 2013	9	295,941	0.30 per 10,000 pregnancies^a^	(0.16–0.58)	Pandemic	Case series	NOS = 8	Both	13 months	US
Knight 2011	59	86,449	6.8 per 10,000 pregnancies	(5.2–8.8)	Pandemic	Case series	JBI = 6/7	Hospital	3 months	Australia/New Zealand
	57	332,569	1.7 per 10,000 pregnancies	(1.3–2.2)					5 months	UK
	Peak: 51	193,796	2.6 per 10,000 pregnancies	(2.0–3.5)					3 months	UK

^a^Calculated from provided number of cases or %

^b^ *NOS* Newcastle-Ottawa Scale, *JBI* JBI Critical Appraisal Checklist for Descriptive/Case Series, *Cochrane* The Cochrane Collaboration’s tool for assessing risk of bias in randomised trials

**Table 6:** Summary of studies reporting incidence of influenza-associated deaths

Study	Number of cases	Denominator	Crude incidence rate (CI)	95% CI	Pandemic or seasonal?	Study design	Grading system and score^b^	Hospital-based or clinic-based?	Inclusion criteria	Duration of surveillance (months or years)	Country
Jamieson 2009	1	3,392,060	0.003 per 10,000 pregnancies^a^	(0.000–0.021)	Pandemic	Case series	JBI = 6/7	Both	Pregnant women with laboratory-confirmed (PCR or culture) H1N1	1 month	US
Creanga 2010	2	113,877	0.18 per 10,000 pregnancies	(0.04–0.70)	Pandemic	Case series	JBI = 6/7	Hospital	Reproductive-aged women with rtPCR-confirmed H1N1 hospitalized in NYC	2 months	US
Knight 2011	6 4	86,449 193,796	0.69 per 10,000 pregnancies 0.21 per 10,000 pregnancies^b^	(0.26–1.51) (0.06–0.53)	Pandemic	Case series	JBI = 6/7	Hospital	Confirmed AH1N1v infection during pregnancy and subsequently admitted to ICU	5 months 3 months	Australia/New Zealand UK
Doyle 2013	8	295,941	0.27 per 10,000 pregnancies^a^	(0.14–0.54)	Pandemic	Case series	NOS = 8	Both	Acute respiratory illness, with laboratory confirmation of pH1N1 by a certified laboratory	13 months	US

^a^Calculated from provided number of cases or %

^b^ *NOS* Newcastle-Ottawa Scale, *JBI* JBI Critical Appraisal Checklist for Descriptive/Case Series

## Discussion

### Main findings

In our systematic review of the scientific literature, we found few studies that described incidence rates of laboratory-confirmed influenza-associated outcomes in pregnant women. We identified a maximum of four studies for each of the outcomes of interest, and through an evaluation that used the GRADE criteria, we rated the overall quality of the evidence to be very low for all outcomes. The Madhi et al. study [5] was the only randomized controlled trial, and this had high quality data, while the other eight articles were based on cohort studies and case series and were considered to be of lower quality. Only three articles [5, 28, 30] described seasonal influenza, and two of these studies [28, 30] included information mostly on subclinical infection. Nearly all studies (5 out of 6) that reported symptomatic infection, hospitalizations, ICU admissions and death described data from 2009 pandemic (H1N1) influenza. There were no data from low-income countries.

The pandemic influenza studies, while more numerous, varied considerably in their approaches to case ascertainment. In particular, the three studies from the United States that described rates of 2009 pandemic (H1N1) influenza-associated outcomes among pregnant women during the pandemic relied on provider initiative to send samples from suspected cases for testing, [26, 31, 32] and also required that public health laboratories report cases to the city [31] the state [26] or the US Centers for Disease Control [32]. For this reason, the number of reported cases is likely a substantial underestimation of the true number of cases in these studies. The variability in surveillance for laboratory-confirmed influenza during the pandemic was likely in part due to the limited availability initially of laboratory testing, which is a challenge inherent to pandemics of novel viruses.

Additionally, studies reporting results from the 2009 influenza A (H1N1) pandemic were conducted at different time periods during the pandemic. Two studies captured less than two months of surveillance, [31, 32] and the studies from the United Kingdom captured only the second wave of the pandemic [25, 27]. Differences in timing and duration of surveillance periods make comparisons challenging. For example, previous studies have shown that the first and second waves of the 2009 pandemic varied considerably in many countries [34–36]. Further, incidence rates of illness during the influenza season are likely to be substantially higher than incidence rates of illness observed over an entire calendar year.

### Interpretation

We limited our search to studies with laboratory-confirmed influenza virus infection, which may have underestimated case rates because of variable testing and reporting of results. Other studies have reported estimated incidence rates of influenza-associated outcomes among pregnant women without laboratory confirmation of influenza disease. A study that used multiple data sources-not all of which contained laboratory-confirmed influenza disease-to quantify influenza-associated hospitalizations in Canada from 1994–2000 reported a rate of 150 influenza-associated hospitalizations (95% CI: 140–170) per 100,000 pregnant women per influenza season [37]. This rate was more than 10-fold higher than the highest rate reported by the four studies of laboratory-confirmed influenza hospitalizations among pregnant women in our study. A study of symptomatic probable and confirmed cases of 2009 pandemic influenza among pregnant women on Reunion Island found a rate of 3,568 cases per 100,000 pregnant women (95% CI: 3,267–3,868),[38] an incidence similar to the highest rates of symptomatic influenza reported by the Madhi et al. study in our review [5]. While these two studies may have overestimated the number of cases given their lack of laboratory confirmation, they provide an upper range of incidence for comparison.

Other studies that did not rely exclusively on laboratory-confirmed influenza virus infection have found rates of influenza-associated deaths in pregnancy that were similar to those that we found. A recent publication on confirmed or possible 2009 influenza A (H1N1) influenza virus infection from April to June 2009 in the United States [39] reported a rate of 2.2 influenza-associated maternal deaths per 100,000 live births, which was in the range of 3 of the 4 studies reported in our review, all of which had wide confidence intervals. A study that used diagnostic billing (ICD-9) codes to determine influenza-associated deaths among pregnant women during the influenza season from 1998 to 2005 found rates that ranged from 0.01 deaths per 10,000 live births to 0.059 deaths per 10,000 live births per influenza season,[40] which corresponded with the low end of reported incidence rates from the four pandemic influenza studies in our review.

Studies are lacking that describe rates of laboratory-confirmed influenza in non-pregnant women of childbearing age, which makes it difficult to assess pregnancy as a risk factor for severe influenza illness. A study from the United States that evaluated rates of influenza-attributable hospitalizations for and deaths from pneumonia, influenza, and other selected acute cardiopulmonary conditions (identified by ICD-9 codes) found that among non-pregnant women aged 15 to 44 without high-risk conditions, an estimated 4 influenza-attributable hospitalizations and deaths (combined) per 10,000 person-years occurred. Among women aged 45 to 64 years without high-risk comorbidities, 6 influenza-attributable hospitalizations and deaths (combined) per 10,000 person-years occurred [41]. A modelling study from South Africa estimated mean annual seasonal influenza-associated mortality to be 0.9 per 100,000 person-years among HIV-negative non-pregnant women. The estimated mean annual influenza-associated mortality in 2009, when the pandemic circulated, was 9.4 per 100,000 person-years among HIV-negative non-pregnant women [42]. In comparison, the rates for hospitalizations associated with laboratory-confirmed influenza among pregnant women described in the articles in our literature review were in a similar range to the rates for non-pregnant women in the US study. The mortality rates for pandemic influenza in the studies that we included were slightly lower than the estimated rate in the South African study for non-pregnant women. However, these comparisons are considerably limited by the differences in how cases were defined. In addition, the US studies described seasonal influenza only, while all four studies included in our review described rates associated with pandemic influenza.

### Strengths and limitations

For this report, we employed a comprehensive literature search strategy and assessed the quality of the evidence for each article; both are strengths of the study. We had a small number of studies for each outcome, with the exception of DALYs, for which we did not identify any studies. We found very few relevant studies that described seasonal influenza, no studies from a low-income country, and only one study from a middle-income country. The diverse methodologies, particularly regarding case ascertainment, and the variable durations of the studies made it methodologically inappropriate to pool results across studies for any of the outcomes. Measures of disease according to severity may have been affected by contextual factors. Decisions to seek care as an outpatient for influenza-like symptoms, and decisions to admit patients to a hospital and to an ICU may vary geographically [25]. Furthermore, decisions to test patients, when not standardized, likely vary by physician, region, and country. In addition, while we identified events associated with influenza virus infection, we were unable to discern events that were causally linked to influenza virus infection. Pregnant women may have complications of influenza virus infection but have no evidence of influenza at the time of hospitalization, and others may have coincidental influenza virus infection that is unrelated to their primary disease process.

Our literature review did not capture grey literature, including unpublished reports of influenza surveillance in pregnant women. While some unpublished surveillance reports describe important trends in the outcomes of interest in this study, denominator data may be lacking,[43] and influenza cases in these reports are often not confirmed by laboratory testing [44]. Therefore, many of these reports would not likely have met our study’s inclusion criteria.

Additionally, there are no consensus scoring tools to assess quality of the included influenza surveillance studies. The Newcastle-Ottawa Scale, the Cochrane Collaboration Risk of Bias Tool, the JBI Critical Appraisal Checklist for Descriptive/Case Series, and GRADE are all helpful tools for evaluation data quality, but they may not have identified all of the quality elements of included studies.

## Conclusions

The goal of this study was to estimate rates of severe laboratory-confirmed influenza disease in pregnant women in order to inform public health decision-making. We found a limited number of studies describing influenza incidence in pregnancy, and these studies employed heterogeneous methodologies. In light of these findings, we suggest that caution be taken when using data generated by our review to estimate disease activity or vaccine program impact. WHO influenza immunization policy recommendations, including the current recommendation that countries considering the initiation or expansion of programmes for seasonal influenza vaccination should prioritize pregnant women over other high-risk groups [1], are made to assist countries with identification of priority groups based not only on disease burden, but also on other factors, including vaccine performance and programmatic opportunities. Pregnant women have been identified as having increased risk and incidence of severe influenza disease by a number of studies that did not meet inclusion criteria for our review. These studies, however, include designs that are generally considered lower quality in the hierarchy of evidence: studies that lack laboratory confirmation, lack population denominators, or use ecological study methods. However, our study demonstrates that the incidence rate of severe disease in pregnant women remains difficult to quantify. More studies with consistent, high-quality surveillance methods and powered to be able to identify less common occurrences like ICU admissions and deaths would be helpful in better understanding the incidence of outcomes associated with influenza virus infection in pregnancy.

## Additional files

Additional file 1: Final Search Strategies For Systematic Literature Review of Incidence of Influenza Virus Infection among Pregnant Women. (DOCX 42 kb)

Additional file 2: Application of GRADE evaluation to various outcomes of systematic literature review of the incidence of influenza virus infection among pregnant women. (DOCX 151 kb)

## Abbreviations

DALY: Disability adjusted life year
GRADE: The grading of recommendations assessment, development and evaluation
WHO: World health organization

## References

[1] WHO | 23 November 2012, vol. 87, 47 (pp. 461–476) n.d

[2] Annual flu programme - GOV.UK n.d. https://www.gov.uk/government/collections/annual-flu-programme (accessed Dec. 8, 2015)

[3] Prevention and control of seasonal influenza with vaccines (2013). Recommendations of the advisory committee on immunization practices-United States, 2013–2014. MMWR Recomm Rep.

[4] Prevention and Control of Influenza n.d. http://www.cdc.gov/mmwr/preview/mmwrhtml/rr5208a1.htm (accessed Dec. 8, 2015)

[5] Madhi SA, Cutland CL, Kuwanda L, Weinberg A, Hugo A, Jones S (2014). Influenza vaccination of pregnant women and protection of their infants. N Engl J Med.

[6] Thompson MG, Li D-K, Shifflett P, Sokolow LZ, Ferber JR, Kurosky S (2014). Effectiveness of seasonal trivalent influenza vaccine for preventing influenza virus illness among pregnant women: a population-based case-control study during the 2010–2011 and 2011–2012 influenza seasons. Clin Infect Dis.

[7] Eick AA, Uyeki TM, Klimov A, Hall H, Reid R, Santosham M (2011). Maternal influenza vaccination and effect on influenza virus infection in young infants. Arch Pediatr Adolesc Med.

[8] Zaman K, Roy E, Arifeen SE, Rahman M, Raqib R, Wilson E (2008). Effectiveness of maternal influenza immunization in mothers and infants. N Engl J Med.

[9] Lambach P, Hombach J, Ortiz JR (2015). A global perspective of maternal influenza immunization. Vaccine.

[10] WHO | WHO Taskforce to evaluate influenza data to in form vaccine impact and economic modelling n.d. http://www.who.int/immunization/research/meetings_workshops/taskforceinterimreportMarch2015/en/.

[11] Moher D, Liberati A, Tetzlaff J, Altman DG. Preferred reporting items for systematic reviews and meta-analyses: the PRISMA statement. BMJ. 2009;339:b2535. 10.1136/bmj.b2535 PMC2714657 19622551

[12] Rathbone J, Hoffmann T, Glasziou P (2015). Faster title and abstract screening? evaluating abstrackr, a semi-automated online screening program for systematic reviewers. Syst Rev.

[13] WHO | Standardization of terminology of the pandemic<br>A (H1N1) 2009 virus n.d. 22046596

[14] McPheeters ML, Kripalani S, Peterson NB, Idowu RT, Jerome RN, Potter SA, et al. Thresholds for Quality Assessment n.d PMC4781280 24422952

[15] Higgins JPT, Altman DG, Gøtzsche PC, Jüni P, Moher D, Oxman AD (2011). The Cochrane Collaboration’s tool for assessing risk of bias in randomised trials. BMJ.

[16] Munn Z, Moola S, Riitano D, Lisy K (2014). The development of a critical appraisal tool for use in systematic reviews addressing questions of prevalence. Int J Heal Policy Manag.

[17] Huguet A, Hayden JA, Stinson J, McGrath PJ, Chambers CT, Tougas ME (2013). Judging the quality of evidence in reviews of prognostic factor research: adapting the GRADE framework. Syst Rev.

[18] Guyatt G, Oxman AD, Akl EA, Kunz R, Vist G, Brozek J (2011). GRADE guidelines: 1. Introduction-GRADE evidence profiles and summary of findings tables. J Clin Epidemiol.

[19] Freeman M, Tukey J (1950). Transformations related to the angular and the square root. Ann Math Stat.

[20] Nyaga VN, Arbyn M, Aerts M (2014). Metaprop : a Stata command to perform meta-analysis of binomial data. Arch Public Heal.

[21] Higgins JPT, Thompson SG (2002). Quantifying heterogeneity in a meta-analysis. Stat Med.

[22] Wickham H (2009). ggplot2: elegant graphics for data analysis.

[23] Higgins JPT, Thompson SG, Deeks JJ, Altman DG (2003). Measuring inconsistency in meta-analyses. BMJ.

[24] Webb SAR, Pettilä V, Seppelt I, Bellomo R, Bailey M, Cooper DJ (2009). Critical care services and 2009 H1N1 influenza in Australia and New Zealand. N Engl J Med.

[25] Knight M, Pierce M, Seppelt I, Kurinczuk JJ, Spark P, Brocklehurst P (2011). Critical illness with AH1N1v influenza in pregnancy: a comparison of two population-based cohorts. BJOG.

[26] Doyle TJ, Goodin K, Hamilton JJ (2013). Maternal and neonatal outcomes among pregnant women with 2009 pandemic influenza a (H1N1) illness in Florida, 2009–2010: a population-based Cohort study. PLoS One.

[27] Yates L, Pierce M, Stephens S, Mill AC, Spark P, Kurinczuk JJ (2010). Influenza A/H1N1v in pregnancy: an investigation of the characteristics and management of affected women and the relationship to pregnancy outcomes for mother and infant. Health Technol Assess.

[28] Griffiths PD, Ronalds CJ, Heath RB (1980). A prospective study of influenza infections during pregnancy. J Epidemiol Community Health.

[29] HARDY JM, AZAROWICZ EN, MANNINI A, MEDEARIS DN, COOKE RE (1961). The effect of Asian influenza on the outcome of pregnancy, Baltimore, 1957–1958. Am J Public Health Nations Health.

[30] Irving WL, James DK, Stephenson T, Laing P, Jameson C, Oxford JS (2000). Influenza virus infection in the second and third trimesters of pregnancy: a clinical and seroepidemiological study. BJOG.

[31] Creanga AA, Johnson TF, Graitcer SB, Hartman LK, Al-Samarrai T, Schwarz AG (2010). Severity of 2009 pandemic influenza A (H1N1) virus infection in pregnant women. Obstet Gynecol.

[32] Jamieson DJ, Honein MA, Rasmussen SA, Williams JL, Swerdlow DL, Biggerstaff MS (2009). H1N1 2009 influenza virus infection during pregnancy in the USA. Lancet.

[33] South Africa | Data, The World Bank n.d. http://data.worldbank.org/country/south-africa (accessed Jan. 4, 2016).

[34] Mytton OT, Rutter PD, Mak M, Stanton EAI, Sachedina N, Donaldson LJ (2012). Mortality due to pandemic (H1N1) 2009 influenza in England: a comparison of the first and second waves. Epidemiol Infect.

[35] team EC for DP and C (ECDC)-HCU-E editorial. The second wave of 2009 pandemic influenza A (H1N1) in New Zealand, January–October 2010 2011. 21329643

[36] Mummert A, Weiss H, Long L-P, Amigó JM, Wan X-F (2013). A perspective on multiple waves of influenza pandemics. PLoS One.

[37] Schanzer DL, Langley JM, Tam TWS (2007). Influenza-attributed hospitalization rates among pregnant women in Canada 1994–2000. J Obstet Gynaecol Can.

[38] Gérardin P, El Amrani R, Cyrille B, Gabrièle M, Guillermin P, Boukerrou M (2010). Low clinical burden of 2009 pandemic influenza A (H1N1) infection during pregnancy on the island of La Réunion. PLoS One.

[39] Callaghan WM, Creanga AA, Jamieson DJ (2015). Pregnancy-related mortality resulting from influenza in the United States during the 2009–2010 pandemic. Obstet Gynecol.

[40] Callaghan WM, Chu SY, Jamieson DJ (2010). Deaths from seasonal influenza among pregnant women in the United States, 1998–2005. Obstet Gynecol.

[41] Neuzil KM, Reed GW, Mitchel EF, Simonsen L, Griffin MR (1998). Impact of influenza on acute cardiopulmonary hospitalizations in pregnant women. Am J Epidemiol.

[42] Tempia S, Walaza S, Cohen AL, von Mollendorf C, Moyes J, McAnerney JM (2015). Mortality associated with seasonal and pandemic influenza among pregnant and nonpregnant women of childbearing Age in a high-HIV-prevalence setting-South Africa, 1999–2009. Clin Infect Dis.

[43] grippenet.fr: Devenez acteur de la surveillance de la grippe n.d. https://www.grippenet.fr/(accessed Feb. 10, 2016).

[44] Knight M, Kenyon S, Brocklehurst P, Neilson J, Shakespeare J, Kurinczuk JJ (Eds.) on behalf of MBRRACEUK. Saving Lives, Improving Mothers’ Care-Lessons learned to inform future maternity care from the UK and Ireland Confidential Enquiries into Maternal Deaths and Morbidity 2009–12. Oxford: National Perinatal Epidemiology Unit, University of Oxford; 2014.

