# Erratum to: Incidence of influenza virus infection among pregnant women: a systematic review

**DOI:** 10.1186/s12884-017-1387-4

**Published:** 2017-06-19

**Authors:** Mark A. Katz, Bradford D. Gessner, Jeanene Johnson, Becky Skidmore, Marian Knight, Niranjan Bhat, Helen Marshall, David J. Horne, Justin R. Ortiz, Deshayne B. Fell

**Affiliations:** 1 Department of Health Systems Management, Medical School for International Health, Ben Gurion University of the Negev, Beer Sheva, Israel; 2 University of Michigan School of Public Health, Ann Arbor, MI USA; 3 Agence de Médecine Préventive, Paris, France; 4 Independent consultant, Oakland, USA; 5 Independent consultant, Ottawa, Canada; 6 National Perinatal Epidemiology Unit (NPEU), University of Oxford, Oxford, UK; 7 Vaccine Access and Delivery Program, PATH, Seattle, WA USA; 8 Vaccinology and Immunology Research Trials Unit, Discipline of Paediatrics, Women’s and Children’s Hospital and University of Adelaide, Adelaide, Australia; 9 Robinson Research Institute, University of Adelaide, North Adelaide, Australia; 10 Department of Medicine, University of Washington, Seattle, USA; 11 Initiative for Vaccine Research, World Health Organization, Geneva, Switzerland; 12 Better Outcomes Registry & Network (BORN), CHEO Research Institute, Ottawa, Canada; 13 Department of Epidemiology, Biostatistics and Occupational Health, McGill University, Montreal, Canada; 14 Agence de Médecine Préventive, Anchorage, AK USA; 15 Los Gatos, CA USA; 16 Harborview Medical Center, University of Washington, Seattle, WA USA

## Erratum

Upon publication of the original article [1], it was noticed that the Acknowledgements Section was missing. The acknowledgements should read as, “The authors would like to thank the following individuals for helpful comments on an earlier version of the study manuscript: David Savitz, Julien Beauté, Pasi Penttinen, and Kevin Pottie. In addition, the authors would like to acknowledge the contributions of the Centers for Disease Control and Prevention (CDC), which provides financial support to the World Health Organization Initiative for Vaccine Research (U50 CK000431).” This has now been acknowledged and corrected in this erratum.
