# Supplementary material for: Incidence of influenza virus infection among pregnant women: a systematic review
Source: BMC Pregnancy Childbirth. 2017 May 30;17:155. doi: 10.1186/s12884-017-1333-5 (PMC5450114; doi:10.1186/s12884-017-1333-5)
Supplement: Supplementary file 1 — Final Search Strategies For Systematic Literature Review of Incidence of Influenza Virus Infection among Pregnant Women. (DOCX 42 kb) [file 12884_2017_1333_MOESM1_ESM.docx]

Appendix A. Final Search Strategies For Systematic Literature Review of Incidence of Influenza Virus Infection among Pregnant Women

20 February 2015

| PubMed  Search | Query | Items found |
| --- | --- | --- |
| [#65](http://www.ncbi.nlm.nih.gov/pubmed/advanced) | Search #63 NOT #64 | [569](http://www.ncbi.nlm.nih.gov/pubmed/?cmd=HistorySearch&querykey=65) |
| [#64](http://www.ncbi.nlm.nih.gov/pubmed/advanced) | Search comment [pt] OR editorial [pt] OR interview [pt] | [888759](http://www.ncbi.nlm.nih.gov/pubmed/?cmd=HistorySearch&querykey=64) |
| [#63](http://www.ncbi.nlm.nih.gov/pubmed/advanced) | Search #61 NOT #62 | [573](http://www.ncbi.nlm.nih.gov/pubmed/?cmd=HistorySearch&querykey=63) |
| [#62](http://www.ncbi.nlm.nih.gov/pubmed/advanced) | Search Animals [mesh] NOT (Animals [mesh] AND Humans [mesh]) | [3976159](http://www.ncbi.nlm.nih.gov/pubmed/?cmd=HistorySearch&querykey=62) |
| [#61](http://www.ncbi.nlm.nih.gov/pubmed/advanced) | Search #31 OR #38 OR #50 OR #60 | [588](http://www.ncbi.nlm.nih.gov/pubmed/?cmd=HistorySearch&querykey=61) |
| [#60](http://www.ncbi.nlm.nih.gov/pubmed/advanced) | Search #23 AND #59 | [482](http://www.ncbi.nlm.nih.gov/pubmed/?cmd=HistorySearch&querykey=60) |
| [#59](http://www.ncbi.nlm.nih.gov/pubmed/advanced) | Search #51 OR #52 OR #53 OR #54 OR #55 OR #56 OR #57 OR #58 | [1986473](http://www.ncbi.nlm.nih.gov/pubmed/?cmd=HistorySearch&querykey=59) |
| [#58](http://www.ncbi.nlm.nih.gov/pubmed/advanced) | Search ecological study [tw] OR ecological studies [tw] | [2999](http://www.ncbi.nlm.nih.gov/pubmed/?cmd=HistorySearch&querykey=58) |
| [#57](http://www.ncbi.nlm.nih.gov/pubmed/advanced) | Search case-control [tw] OR case-base [tw] OR case-based [tw] OR case-comparison [tw] OR case-compeer [tw] OR case-referent [tw] OR case-referrent [tw] | [221179](http://www.ncbi.nlm.nih.gov/pubmed/?cmd=HistorySearch&querykey=57) |
| [#56](http://www.ncbi.nlm.nih.gov/pubmed/advanced) | Search Case-Control Studies [mesh] | [690148](http://www.ncbi.nlm.nih.gov/pubmed/?cmd=HistorySearch&querykey=56) |
| [#55](http://www.ncbi.nlm.nih.gov/pubmed/advanced) | Search population study [tw] OR population studies [tw] OR population-based study [tw] OR population-based studies [tw] OR population analys* [tw] OR population-based analys* [tw] | [34776](http://www.ncbi.nlm.nih.gov/pubmed/?cmd=HistorySearch&querykey=55) |
| [#54](http://www.ncbi.nlm.nih.gov/pubmed/advanced) | Search followup study [tw] OR followup studies [tw] OR follow up study [tw] OR follow up studies [tw] | [523417](http://www.ncbi.nlm.nih.gov/pubmed/?cmd=HistorySearch&querykey=54) |
| [#53](http://www.ncbi.nlm.nih.gov/pubmed/advanced) | Search longitudinal [tw] OR prospective [tw] OR retrospective [tw] | [1272197](http://www.ncbi.nlm.nih.gov/pubmed/?cmd=HistorySearch&querykey=53) |
| [#52](http://www.ncbi.nlm.nih.gov/pubmed/advanced) | Search cohort [w] OR cohorts [tw] | [374244](http://www.ncbi.nlm.nih.gov/pubmed/?cmd=HistorySearch&querykey=52) |
| [#51](http://www.ncbi.nlm.nih.gov/pubmed/advanced) | Search Cohort Studies [mesh] | [1389589](http://www.ncbi.nlm.nih.gov/pubmed/?cmd=HistorySearch&querykey=51) |
| [#50](http://www.ncbi.nlm.nih.gov/pubmed/advanced) | Search #23 AND #49 | [34](http://www.ncbi.nlm.nih.gov/pubmed/?cmd=HistorySearch&querykey=50) |
| [#49](http://www.ncbi.nlm.nih.gov/pubmed/advanced) | Search #39 OR #40 OR #41 OR #42 OR #43 OR #44 OR #45 OR #46 OR #47 OR #48 | [384409](http://www.ncbi.nlm.nih.gov/pubmed/?cmd=HistorySearch&querykey=49) |
| [#48](http://www.ncbi.nlm.nih.gov/pubmed/advanced) | Search control group [tw] OR control groups [tw] | [308133](http://www.ncbi.nlm.nih.gov/pubmed/?cmd=HistorySearch&querykey=48) |
| [#47](http://www.ncbi.nlm.nih.gov/pubmed/advanced) | Search Control Groups [mesh] | [1432](http://www.ncbi.nlm.nih.gov/pubmed/?cmd=HistorySearch&querykey=47) |
| [#46](http://www.ncbi.nlm.nih.gov/pubmed/advanced) | Search interrupted time series [tw] | [1170](http://www.ncbi.nlm.nih.gov/pubmed/?cmd=HistorySearch&querykey=46) |
| [#45](http://www.ncbi.nlm.nih.gov/pubmed/advanced) | Search Interrupted Time Series Analysis [mesh] | [16](http://www.ncbi.nlm.nih.gov/pubmed/?cmd=HistorySearch&querykey=45) |
| [#44](http://www.ncbi.nlm.nih.gov/pubmed/advanced) | Search controlled study [tw] OR controlled studies [tw] | [48704](http://www.ncbi.nlm.nih.gov/pubmed/?cmd=HistorySearch&querykey=44) |
| [#43](http://www.ncbi.nlm.nih.gov/pubmed/advanced) | Search Historically Controlled Study [mesh] | [6](http://www.ncbi.nlm.nih.gov/pubmed/?cmd=HistorySearch&querykey=43) |
| [#42](http://www.ncbi.nlm.nih.gov/pubmed/advanced) | Search "controlled before and after" [tw] OR "controlled before after" [tw] | [576](http://www.ncbi.nlm.nih.gov/pubmed/?cmd=HistorySearch&querykey=42) |
| [#41](http://www.ncbi.nlm.nih.gov/pubmed/advanced) | Search Controlled Before-After Studies [mesh] | [23](http://www.ncbi.nlm.nih.gov/pubmed/?cmd=HistorySearch&querykey=41) |
| [#40](http://www.ncbi.nlm.nih.gov/pubmed/advanced) | Search nRCT [tw] OR nRCTs [tw] OR non-RCT [tw] OR non-RCTs [tw] | [338](http://www.ncbi.nlm.nih.gov/pubmed/?cmd=HistorySearch&querykey=40) |
| [#39](http://www.ncbi.nlm.nih.gov/pubmed/advanced) | Search nonrandom* [tw] OR non-random* [tw] OR quasi-random* [tw] OR quasi-experiment* [tw] | [36211](http://www.ncbi.nlm.nih.gov/pubmed/?cmd=HistorySearch&querykey=39) |
| [#38](http://www.ncbi.nlm.nih.gov/pubmed/advanced) | Search #23 AND #37 | [75](http://www.ncbi.nlm.nih.gov/pubmed/?cmd=HistorySearch&querykey=38) |
| [#37](http://www.ncbi.nlm.nih.gov/pubmed/advanced) | Search #32 OR #33 OR #34 OR #35 OR #36 | [1047396](http://www.ncbi.nlm.nih.gov/pubmed/?cmd=HistorySearch&querykey=37) |
| [#36](http://www.ncbi.nlm.nih.gov/pubmed/advanced) | Search trial [ti] | [134771](http://www.ncbi.nlm.nih.gov/pubmed/?cmd=HistorySearch&querykey=36) |
| [#35](http://www.ncbi.nlm.nih.gov/pubmed/advanced) | Search single blind* [tw] OR double blind* [tw] OR triple blind* [tw] OR single mask* [tw] OR double mask* [tw] OR triple mask* [tw] OR single dumm* [tw] OR double dumm* [tw] OR triple dumm* [tw] | [184121](http://www.ncbi.nlm.nih.gov/pubmed/?cmd=HistorySearch&querykey=35) |
| [#34](http://www.ncbi.nlm.nih.gov/pubmed/advanced) | Search randomised [tw] OR randomized [tw] OR randomly [tw] OR RCT [tw] OR RCTs [tw] OR placebo* [tw] | [805402](http://www.ncbi.nlm.nih.gov/pubmed/?cmd=HistorySearch&querykey=34) |
| [#33](http://www.ncbi.nlm.nih.gov/pubmed/advanced) | Search "clinical trials as topic" [mesh] | [282821](http://www.ncbi.nlm.nih.gov/pubmed/?cmd=HistorySearch&querykey=33) |
| [#32](http://www.ncbi.nlm.nih.gov/pubmed/advanced) | Search controlled clinical trial [pt] OR randomized controlled trial [pt] | [465939](http://www.ncbi.nlm.nih.gov/pubmed/?cmd=HistorySearch&querykey=32) |
| [#31](http://www.ncbi.nlm.nih.gov/pubmed/advanced) | Search #23 AND #30 | [67](http://www.ncbi.nlm.nih.gov/pubmed/?cmd=HistorySearch&querykey=31) |
| [#30](http://www.ncbi.nlm.nih.gov/pubmed/advanced) | Search #24 OR #25 OR #26 OR #27 OR #28 OR #29 | [264499](http://www.ncbi.nlm.nih.gov/pubmed/?cmd=HistorySearch&querykey=30) |
| [#29](http://www.ncbi.nlm.nih.gov/pubmed/advanced) | Search "The Cochrane database of systematic reviews"[Journal] OR "evidence report/technology assessment summary"[Journal] OR "evidence report/technology assessment"[Journal] | [11002](http://www.ncbi.nlm.nih.gov/pubmed/?cmd=HistorySearch&querykey=29) |
| [#28](http://www.ncbi.nlm.nih.gov/pubmed/advanced) | Search meta-review* [tw] OR meta-overview* [tw] OR meta-synthes* [tw] OR "review of reviews" [tw] | [481](http://www.ncbi.nlm.nih.gov/pubmed/?cmd=HistorySearch&querykey=28) |
| [#27](http://www.ncbi.nlm.nih.gov/pubmed/advanced) | Search meta-analy* [tw] OR metanaly* [tw] OR metaanaly* [tw] OR met analy* [tw] OR integrative research [tw] OR integrative review* [tw] OR integrative overview* [tw] OR research integration [tw] OR research overview* [tw] OR collaborative review* [tw] | [96927](http://www.ncbi.nlm.nih.gov/pubmed/?cmd=HistorySearch&querykey=27) |
| [#26](http://www.ncbi.nlm.nih.gov/pubmed/advanced) | Search "meta-analysis as topic" [mesh] | [13791](http://www.ncbi.nlm.nih.gov/pubmed/?cmd=HistorySearch&querykey=26) |
| [#25](http://www.ncbi.nlm.nih.gov/pubmed/advanced) | Search meta analysis [pt] | [52097](http://www.ncbi.nlm.nih.gov/pubmed/?cmd=HistorySearch&querykey=25) |
| [#24](http://www.ncbi.nlm.nih.gov/pubmed/advanced) | Search systematic [sb] | [241380](http://www.ncbi.nlm.nih.gov/pubmed/?cmd=HistorySearch&querykey=24) |
| [#23](http://www.ncbi.nlm.nih.gov/pubmed/advanced) | Search #13 AND #22 | [1926](http://www.ncbi.nlm.nih.gov/pubmed/?cmd=HistorySearch&querykey=23) |
| [#22](http://www.ncbi.nlm.nih.gov/pubmed/advanced) | Search #14 OR #15 OR #16 OR #17 OR #18 OR #19 OR #20 OR #21 | [5237106](http://www.ncbi.nlm.nih.gov/pubmed/?cmd=HistorySearch&querykey=22) |
| [#21](http://www.ncbi.nlm.nih.gov/pubmed/advanced) | Search Influenza, Human/ep [mesh] | [15073](http://www.ncbi.nlm.nih.gov/pubmed/?cmd=HistorySearch&querykey=21) |
| [#20](http://www.ncbi.nlm.nih.gov/pubmed/advanced) | Search burden [tw] OR burdens [tw] OR death [tw] OR deaths [tw] OR epidemiolog* [tw] OR incidence [tw] OR frequenc* [tw] OR morbidit* [tw] OR mortalit* [tw] OR occurrence* [tw] OR occurence* [tw] OR outbreak* [tw] OR prevalen* [tw] OR rate [tw] OR rates [tw] OR surveillance* [tw] | [5197551](http://www.ncbi.nlm.nih.gov/pubmed/?cmd=HistorySearch&querykey=20) |
| [#19](http://www.ncbi.nlm.nih.gov/pubmed/advanced) | Search Cause of Death [mesh] | [36044](http://www.ncbi.nlm.nih.gov/pubmed/?cmd=HistorySearch&querykey=19) |
| [#18](http://www.ncbi.nlm.nih.gov/pubmed/advanced) | Search Hospital Mortality [mesh] | [24126](http://www.ncbi.nlm.nih.gov/pubmed/?cmd=HistorySearch&querykey=18) |
| [#17](http://www.ncbi.nlm.nih.gov/pubmed/advanced) | Search Mortality [mesh] | [286173](http://www.ncbi.nlm.nih.gov/pubmed/?cmd=HistorySearch&querykey=17) |
| [#16](http://www.ncbi.nlm.nih.gov/pubmed/advanced) | Search Morbidity [mesh] | [379937](http://www.ncbi.nlm.nih.gov/pubmed/?cmd=HistorySearch&querykey=16) |
| [#15](http://www.ncbi.nlm.nih.gov/pubmed/advanced) | Search Prevalence [mesh] | [196462](http://www.ncbi.nlm.nih.gov/pubmed/?cmd=HistorySearch&querykey=15) |
| [#14](http://www.ncbi.nlm.nih.gov/pubmed/advanced) | Search Incidence [mesh] | [179784](http://www.ncbi.nlm.nih.gov/pubmed/?cmd=HistorySearch&querykey=14) |
| [#13](http://www.ncbi.nlm.nih.gov/pubmed/advanced) | Search #6 AND #12 | [3182](http://www.ncbi.nlm.nih.gov/pubmed/?cmd=HistorySearch&querykey=13) |
| [#12](http://www.ncbi.nlm.nih.gov/pubmed/advanced) | Search #7 OR #8 OR #9 OR #10 OR #11 | [962828](http://www.ncbi.nlm.nih.gov/pubmed/?cmd=HistorySearch&querykey=12) |
| [#11](http://www.ncbi.nlm.nih.gov/pubmed/advanced) | Search maternal* [tw] | [237040](http://www.ncbi.nlm.nih.gov/pubmed/?cmd=HistorySearch&querykey=11) |
| [#10](http://www.ncbi.nlm.nih.gov/pubmed/advanced) | Search prenatal* [tw] OR antenatal* [tw] OR ante natal* [tw] OR antepartum [tw] OR ante partum [tw] OR perinatal* [tw] OR peri natal* [tw] OR peripartum [tw] OR peri partum [tw] | [197952](http://www.ncbi.nlm.nih.gov/pubmed/?cmd=HistorySearch&querykey=10) |
| [#9](http://www.ncbi.nlm.nih.gov/pubmed/advanced) | Search pregnan* [tw] | [804976](http://www.ncbi.nlm.nih.gov/pubmed/?cmd=HistorySearch&querykey=9) |
| [#8](http://www.ncbi.nlm.nih.gov/pubmed/advanced) | Search Pregnancy Complications [mesh] | [344518](http://www.ncbi.nlm.nih.gov/pubmed/?cmd=HistorySearch&querykey=8) |
| [#7](http://www.ncbi.nlm.nih.gov/pubmed/advanced) | Search Pregnancy [mesh] | [720049](http://www.ncbi.nlm.nih.gov/pubmed/?cmd=HistorySearch&querykey=7) |
| [#6](http://www.ncbi.nlm.nih.gov/pubmed/advanced) | Search #1 OR #2 OR #3 OR #4 OR #5 | [105586](http://www.ncbi.nlm.nih.gov/pubmed/?cmd=HistorySearch&querykey=6) |
| [#5](http://www.ncbi.nlm.nih.gov/pubmed/advanced) | Search H1N1 [tw] OR PH1N1 [tw] OR H3N2 [tw] OR AH1N1 [tw] OR AH3N2 [tw] | [17914](http://www.ncbi.nlm.nih.gov/pubmed/?cmd=HistorySearch&querykey=5) |
| [#4](http://www.ncbi.nlm.nih.gov/pubmed/advanced) | Search Influenza B Virus [mesh] | [3065](http://www.ncbi.nlm.nih.gov/pubmed/?cmd=HistorySearch&querykey=4) |
| [#3](http://www.ncbi.nlm.nih.gov/pubmed/advanced) | Search Influenza A Virus [mesh] | [32818](http://www.ncbi.nlm.nih.gov/pubmed/?cmd=HistorySearch&querykey=3) |
| [#2](http://www.ncbi.nlm.nih.gov/pubmed/advanced) | Search influenza* [tw] OR flu [tw] OR grippe [tw] | [105429](http://www.ncbi.nlm.nih.gov/pubmed/?cmd=HistorySearch&querykey=2) |
| [#1](http://www.ncbi.nlm.nih.gov/pubmed/advanced) | Search Influenza, Human [mesh] | [36545](http://www.ncbi.nlm.nih.gov/pubmed/?cmd=HistorySearch&querykey=1) |

Embase

Database: Embase Classic+Embase <1947 to 2015 February 19> Search Strategy:

--------------------------------------------------------------------------------

1 influenza/ (54551)

2 exp Influenza virus A/ (33885)

3 exp Influenza virus B/ (5063)

4 pandemic influenza/ (3527)

5 seasonal influenza/ (3105)

6 (influenza* or flu or grippe).ti,ab,kw. (119616)

7 (H1N1 or PH1N1 or H3N2 or AH1N1 or AH3N2).ti,ab,kw. (18927)

8 or/1-7 (136240)

9 exp pregnancy/ (653194)

10 exp pregnancy complication/ (113301)

11 pregnan*.ti,ab,kw. (525266)

12 (prenatal* or antenatal* or ante natal* or antepartum or ante partum or perinatal* or peri natal* or peripartum or peri partum).ti,ab,kw. (194443)

13 maternal*.ti,ab,kw. (235996)

14 or/9-13 (1015855)

15 8 and 14 (4260)

16 incidence/ (222422)

17 prevalence/ (416473)

18 exp disease surveillance/ (13217)

19 Infection rate/ (18779)

20 maternal morbidity/ (5531)

21 maternal mortality/ (17929)

22 cause of death/ (79544)

23 (burden or burdens or death or deaths or epidemiolog* or incidence or frequenc* or morbidit* or mortalit* or occurrence* or occurence* or outbreak* or prevalen* or rate or rates or surveillance*).ti,ab,kw. (5695929)

24 influenza/ep [Epidemiology] (10297)

25 exp Influenza virus A/ep (200)

26 exp Influenza virus B/ep (18)

27 pandemic influenza/ep (593)

28 seasonal influenza/ep (318)

29 or/16-28 (5836924)

30 15 and 29 (2258)

31 meta-analysis/ (88277)

32 "systematic review"/ (85131)

33 "meta analysis (topic)"/ (17449)

34 (meta-analy* or metanaly* or metaanaly* or met analy* or integrative research or integrative review* or integrative overview* or research integration or research overview* or collaborative review*).ti,ab,kw. (96594)

35 (systematic review* or systematic overview* or evidence-based review* or evidence-based overview* or (evidence adj3 (review* or overview*)) or meta-review* or meta-overview* or meta-synthes* or "review of reviews").ti,ab,kw. (107156)

36 (cochrane or health technology assessment or evidence report).jw. (13030)

37 or/31-36 (234697)

38 30 and 37 (58)

39 randomized controlled trial/ or controlled clinical trial/ (499071)

40 exp "clinical trial (topic)"/ (131988)

41 (randomi#ed or randomly or RCT$1 or placebo*).ti,ab,kw. (863585)

42 ((singl* or doubl* or trebl* or tripl*) adj (mask* or blind* or dumm*)).ti,ab,kw. (175512)

43 trial.ti. (179259)

44 or/39-43 (1223592)

45 30 and 44 (130)

46 (nonrandom* or non-random* or quasi-random* or quasi-experiment*).ti,ab,kw. (42644)

47 (nRCT or nRCTs or non-RCT$1).ti,ab,kw. (440)

48 (control* adj3 ("before and after" or "before after")).ti,ab,kw. (3614)

49 time series analysis/ (15046)

50 (time series adj3 interrupt*).ti,ab,kw. (1343)

51 controlled study/ (4523729)

52 (control* adj2 stud$3).ti,ab,kw. (207283)

53 control group/ (84226)

54 (control$ adj2 group$1).ti,ab,kw. (466190)

55 or/46-54 (4855981)

56 30 and 55 (390)

57 cohort analysis/ (190624)

58 cohort.ti,ab,kw. (412860)

59 retrospective study/ (381545)

60 longitudinal study/ (74058)

61 prospective study/ (277193)

62 (longitudinal or prospective or retrospective).ti,ab,kw. (1085929)

63 follow up/ (906923)

64 ((followup or follow-up) adj (study or studies)).ti,ab,kw. (55551)

65 population research/ (71815)

66 ((population or population-based) adj (study or studies or analys#s)).ti,ab,kw. (16790)

67 exp case control study/ (94264)

68 (case-control* or case-base or case-based or case-comparison or case-compeer or case-referent or case-referrent).ti,ab,kw. (109019)

69 (ecological adj (study or studies)).ti,ab,kw. (3355)

70 or/57-69 (2397764)

71 30 and 70 (583)

72 38 or 45 or 56 or 71 (876)

73 exp animal experimentation/ or exp models animal/ or exp animal experiment/ or nonhuman/ or exp vertebrate/ (21390153)

74 exp humans/ or exp human experimentation/ or exp human experiment/ (15705916)

75 73 not 74 (5685249)

76 72 not 75 (819)

77 (comment or editorial or interview).pt. (466407)

78 76 not 77 (816)

***************************

Cochrane Library

Search Name: Maternal Influenza - Incidence

Date Run: 20/02/15 18:21:54.888

Description: WHO - 2015 Feb 20 - Final

ID Search Hits

#1 [mh "Influenza Human"] 1365

#2 (influenza* or flu or grippe):ti,ab,kw 5505

#3 [mh "Influenza A Virus"] 733

#4 [mh "Influenza B Virus"] 224

#5 (H1N1 or PH1N1 or H3N2 or AH1N1 or AH3N2):ti,ab,kw 818

#6 {or #1-#5} 5508

#7 [mh Pregnancy] 5852

#8 [mh "Pregnancy Complications"] 7900

#9 pregnan*:ti,ab,kw 26916

#10 (prenatal* or antenatal* or (ante next natal*) or antepartum or "ante partum" or perinatal* or (peri next natal*) or peripartum or "peri partum"):ti,ab,kw 6583

#11 maternal*:ti,ab,kw 8827

#12 {or #7-#11} 31907

#13 #6 and #12 101

#14 [mh Incidence] 7910

#15 [mh Prevalence] 3937

#16 [mh Morbidity] 12065

#17 [mh Mortality] 11164

#18 [mh "Hospital Mortality"] 1072

#19 [mh "Cause of Death"] 1182

#20 (burden or burdens or death or deaths or epidemiolog* or frequenc* or incidence or morbidit* or mortalit* or occurrence* or occurence* or outbreak* or prevalen* or rate or rates or surveillance*):ti,ab,kw 272517

#21 [mh "Influenza, Human"/ep] 244

#22 {or #14-#21} 272595

#23 #13 and #22 46

DSR – 3

DARE – 1

CENTRAL – 38

HTA - 1

NHS EED – 3

CINAHL Plus with Full Text

| [Search ID#](javascript:__doPostBack('ctl00$ctl00$FindField$FindField$historyControl$ReorderHistoryLink','')) | Search Terms | Actions |
| --- | --- | --- |
| S64 | S62 NOT S63 | (112) |
| S63 | PT comment OR PT editorial OR PT interview | (211,868) |
| S62 | S60 NOT S61 | (112) |
| S61 | (MH "Animals+") NOT ( (MH "Animals+") AND (MH "Human") ) | (55,337) |
| S60 | S28 OR S35 OR S47 OR S59 | (112) |
| S59 | S22 AND S58 | (91) |
| S58 | S48 OR S49 OR S50 OR S51 OR S52 OR S53 OR S54 OR S55 OR S56 OR S57 | (460,887) |
| S57 | TI ( ecological W1 (study or studies) ) OR AB ( ecological W1 (study or studies) ) | (454) |
| S56 | (MH "Ecological Research") | (589) |
| S55 | TI ( (case W1 control*) or "case-base" or "case-based" or "case-comparison" or "case-compeer" or "case-referent" or "case-referrent" ) OR AB ( (case W1 control*) or "case-base" or "case-based" or "case-comparison" or "case-compeer" or "case-referent" or "case-referrent" ) | (15,349) |
| S54 | (MH "Case Control Studies+") | (50,255) |
| S53 | TI ( (population or "population-based") W1 (study or studies or analys*) ) OR AB ( (population or "population-based") W1 (study or studies or analys*) ) | (12,193) |
| S52 | TI ( (followup or "follow-up") W1 (study or studies) ) OR AB ( (followup or "follow-up") W1 (study or studies) ) | (6,534) |
| S51 | TI ( longitudinal or prospective or retrospective ) OR AB ( longitudinal or prospective or retrospective ) | (143,806) |
| S50 | (MH "Retrospective Design") | (130,960) |
| S49 | TI ( cohort or cohorts ) OR AB ( cohort or cohorts ) | (67,183) |
| S48 | (MH "Prospective Studies+") | (255,496) |
| S47 | S22 AND S46 | (12) |
| S46 | S36 OR S37 OR S38 OR S39 OR S40 OR S41 OR S42 OR S43 OR S44 OR S45 | (86,751) |
| S45 | (MH "Pretest-Posttest Control Group Design") | (424) |
| S44 | TI ( control* N2 (group or groups) ) OR AB ( control* N2 (group or groups) ) | (43,022) |
| S43 | (MH "Control Group") | (6,197) |
| S42 | TI ( control* N2 (study or studies) ) OR AB ( control* N2 (study or studies) ) | (31,770) |
| S41 | TI time series N3 interrupt* OR AB time series N3 interrupt* | (534) |
| S40 | (MH "Quasi-Experimental Studies+") | (8,712) |
| S39 | TI ( control* N3 ("before and after" or "before after") ) OR AB ( control* N3 ("before and after" or "before after") ) | (789) |
| S38 | TI ( nRCT or nRCTs or (non W1 RCT) or (non W1 RCTs) ) OR AB ( nRCT or nRCTs or (non W1 RCT) or (non W1 RCTs) ) | (115) |
| S37 | TI ( nonrandom* or (non W1 random*) or (quasi W1 random*) or (quasi W1 experiment*) ) OR AB ( nonrandom* or (non W1 random*) or (quasi W1 random*) or (quasi W1 experiment*) ) | (9,330) |
| S36 | (MH "Nonrandomized Trials") | (183) |
| S35 | S22 AND S34 | (17) |
| S34 | S29 OR S30 OR S31 OR S32 OR S33 | (253,931) |
| S33 | TI trial | (50,254) |
| S32 | TI ( (singl* or doubl* or trebl* or tripl*) W1 (mask* or blind* or dumm*) ) OR AB ( (singl* or doubl* or trebl* or tripl*) W1 (mask* or blind* or dumm*) ) | (21,337) |
| S31 | TI ( randomized or randomised or randomly or RCT or RCTs or placebo* ) OR AB ( randomized or randomised or randomly or RCT or RCTs or placebo* ) | (136,425) |
| S30 | (MH "Clinical Trials+") | (183,704) |
| S29 | PT randomized controlled trial | (46,029) |
| S28 | S22 AND S27 | (12) |
| S27 | S23 OR S24 OR S25 OR S26 | (73,049) |
| S26 | TI ( (systematic W1 review*) or (systematic W1 overview*) or ("evidence-based" W1 review*) or ("evidence-based" W1 overview*) or (evidence N3 (review* or overview*)) or (meta W1 review*) or (meta W1 overview*) or (meta W1 synthes*) or "review of reviews" ) OR AB ( (systematic W1 review*) or (systematic W1 overview*) or ("evidence-based" W1 review*) or ("evidence-based" W1 overview*) or (evidence N3 (review* or overview*)) or (meta W1 review*) or (meta W1 overview*) or (meta W1 synthes*) or "re [...](javascript:showHistoryTerm('ctl00_ctl00_FindField_FindField_historyControl_HistoryRepeater_ctl38_ellipsis',true)) | (42,813) |
| S25 | TI ( (meta W1 analy*) or metanaly* or metaanaly* or (met W1 analy*) or (integrative W1 research) or (integrative W1 review*) or (integrative W1 overview*) or (research W1 integration) or (research W1 overview*) or (collaborative W1 review*) ) OR AB ( (meta W1 analy*) or metanaly* or metaanaly* or (met W1 analy*) or (integrative W1 research) or (integrative W1 review*) or (integrative W1 overview*) or (research W1 integration) or (research W1 overview*) or (collaborative W1 review*) ) | (24,109) |
| S24 | (MH "Meta Analysis") OR (MH "Meta Synthesis") | (21,748) |
| S23 | (MH "Systematic Review") | (29,898) |
| S22 | S13 AND S21 | (385) |
| S21 | S14 OR S15 OR S16 OR S17 OR S18 OR S19 OR S20 | (536,883) |
| S20 | (MH "Influenza, Human+/EP") | (2,302) |
| S19 | TI ( burden or burdens or death or deaths or epidemiolog* or incidence or frequenc* or morbidit* or mortalit* or occurrence* or occurence* or outbreak* or prevalen* or rate or rates or surveillance* ) OR AB ( burden or burdens or death or deaths or epidemiolog* or incidence or frequenc* or morbidit* or mortalit* or occurrence* or occurence* or outbreak* or prevalen* or rate or rates or surveillance* ) | (491,827) |
| S18 | (MH "Cause of Death") | (7,863) |
| S17 | (MH "Hospital Mortality") | (8,722) |
| S16 | (MH "Maternal Mortality") | (3,369) |
| S15 | MH "Prevalence" | (51,160) |
| S14 | MH "Incidence" | (37,149) |
| S13 | S6 AND S12 | (847) |
| S12 | S7 OR S8 OR S9 OR S10 OR S11 | (163,189) |
| S11 | TI maternal* OR AB maternal* | (30,274) |
| S10 | TI ( prenatal* or antenatal* or (ante W1 natal*) or antepartum or "ante partum" or perinatal* or (peri W1 natal*) or peripartum or "peri partum" ) OR AB ( prenatal* or antenatal* or (ante W1 natal*) or antepartum or "ante partum" or perinatal* or (peri W1 natal*) or peripartum or "peri partum" ) | (27,254) |
| S9 | TI pregnan* OR AB pregnan* | (57,975) |
| S8 | MH "Pregnancy Complications+" | (51,523) |
| S7 | MH "Pregnancy+" | (128,755) |
| S6 | S1 OR S2 OR S3 OR S4 OR S5 | (17,839) |
| S5 | TI ( (H1N1 or PH1N1 or H3N2 or AH1N1 or AH3N2 ) OR AB ( (H1N1 or PH1N1 or H3N2 or AH1N1 or AH3N2 ) | (3,462) |
| S4 | MH "Influenza B Virus" | (184) |
| S3 | MH "Influenza A Virus+" | (3,832) |
| S2 | TI ( influenza* or flu or grippe ) OR AB ( influenza* or flu or grippe ) | (15,738) |
| S1 | MH "Influenza, Human+" | (6,298) |

**Influenza in Pregnancy – Incidence - DALYs**

2015 Feb 28

PubMed

| Search | Query | Items found |
| --- | --- | --- |
| [#15](http://www.ncbi.nlm.nih.gov/pubmed/advanced) | Search #13 AND #14 | [0](http://www.ncbi.nlm.nih.gov/pubmed/?cmd=HistorySearch&querykey=15) |
| [#14](http://www.ncbi.nlm.nih.gov/pubmed/advanced) | Search "disability-adjusted life year" [tw] OR "disability-adjusted life years" [tw] OR DALY [tw] OR DALYs [tw] | [1817](http://www.ncbi.nlm.nih.gov/pubmed/?cmd=HistorySearch&querykey=14) |
| [#13](http://www.ncbi.nlm.nih.gov/pubmed/advanced) | Search #6 AND #12 | [3184](http://www.ncbi.nlm.nih.gov/pubmed/?cmd=HistorySearch&querykey=13) |
| [#12](http://www.ncbi.nlm.nih.gov/pubmed/advanced) | Search #7 OR #8 OR #9 OR #10 OR #11 | [963671](http://www.ncbi.nlm.nih.gov/pubmed/?cmd=HistorySearch&querykey=12) |
| [#11](http://www.ncbi.nlm.nih.gov/pubmed/advanced) | Search maternal* [tw] | [237314](http://www.ncbi.nlm.nih.gov/pubmed/?cmd=HistorySearch&querykey=11) |
| [#10](http://www.ncbi.nlm.nih.gov/pubmed/advanced) | Search prenatal* [tw] OR antenatal* [tw] OR ante natal* [tw] OR antepartum [tw] OR ante partum [tw] OR perinatal* [tw] OR peri natal* [tw] OR peripartum [tw] OR peri partum [tw] | [198184](http://www.ncbi.nlm.nih.gov/pubmed/?cmd=HistorySearch&querykey=10) |
| [#9](http://www.ncbi.nlm.nih.gov/pubmed/advanced) | Search pregnan* [tw] | [805664](http://www.ncbi.nlm.nih.gov/pubmed/?cmd=HistorySearch&querykey=9) |
| [#8](http://www.ncbi.nlm.nih.gov/pubmed/advanced) | Search Pregnancy Complications [mesh] | [344832](http://www.ncbi.nlm.nih.gov/pubmed/?cmd=HistorySearch&querykey=8) |
| [#7](http://www.ncbi.nlm.nih.gov/pubmed/advanced) | Search Pregnancy [mesh] | [720574](http://www.ncbi.nlm.nih.gov/pubmed/?cmd=HistorySearch&querykey=7) |
| [#6](http://www.ncbi.nlm.nih.gov/pubmed/advanced) | Search #1 OR #2 OR #3 OR #4 OR #5 | [105723](http://www.ncbi.nlm.nih.gov/pubmed/?cmd=HistorySearch&querykey=6) |
| [#5](http://www.ncbi.nlm.nih.gov/pubmed/advanced) | Search H1N1 [tw] OR PH1N1 [tw] OR H3N2 [tw] OR AH1N1 [tw] OR AH3N2 [tw] | [17939](http://www.ncbi.nlm.nih.gov/pubmed/?cmd=HistorySearch&querykey=5) |
| [#4](http://www.ncbi.nlm.nih.gov/pubmed/advanced) | Search Influenza B Virus [mesh] | [3067](http://www.ncbi.nlm.nih.gov/pubmed/?cmd=HistorySearch&querykey=4) |
| [#3](http://www.ncbi.nlm.nih.gov/pubmed/advanced) | Search Influenza A Virus [mesh] | [32851](http://www.ncbi.nlm.nih.gov/pubmed/?cmd=HistorySearch&querykey=3) |
| [#2](http://www.ncbi.nlm.nih.gov/pubmed/advanced) | Search influenza* [tw] OR flu [tw] OR grippe [tw] | [105566](http://www.ncbi.nlm.nih.gov/pubmed/?cmd=HistorySearch&querykey=2) |
| [#1](http://www.ncbi.nlm.nih.gov/pubmed/advanced) | Search Influenza, Human [mesh] | [36584](http://www.ncbi.nlm.nih.gov/pubmed/?cmd=HistorySearch&querykey=1) |

Embase

Database: Embase Classic+Embase <1947 to 2015 February 27> Search Strategy:

--------------------------------------------------------------------------------

1 influenza/ (54634)

2 exp Influenza virus A/ (34006)

3 exp Influenza virus B/ (5077)

4 pandemic influenza/ (3549)

5 seasonal influenza/ (3143)

6 (influenza* or flu or grippe).ti,ab,kw. (119886)

7 (H1N1 or PH1N1 or H3N2 or AH1N1 or AH3N2).ti,ab,kw. (18985)

8 or/1-7 (136553)

9 exp pregnancy/ (653951)

10 exp pregnancy complication/ (113430)

11 pregnan*.ti,ab,kw. (526284)

12 (prenatal* or antenatal* or ante natal* or antepartum or ante partum or perinatal* or peri natal* or peripartum or peri partum).ti,ab,kw. (194785)

13 maternal*.ti,ab,kw. (236465)

14 or/9-13 (1017343)

15 8 and 14 (4270)

16 (("disability-adjusted" adj2 year$1) or DALY or DALYs).ti,ab,kw. (2316)

17 15 and 16 (1)

18 meta-analysis/ (88630)

19 "systematic review"/ (85601)

20 "meta analysis (topic)"/ (17638)

21 (meta-analy* or metanaly* or metaanaly* or met analy* or integrative research or integrative review* or integrative overview* or research integration or research overview* or collaborative review*).ti,ab,kw. (97079)

22 (systematic review* or systematic overview* or evidence-based review* or evidence-based overview* or (evidence adj3 (review* or overview*)) or meta-review* or meta-overview* or meta-synthes* or "review of reviews").ti,ab,kw. (107622)

23 (cochrane or health technology assessment or evidence report).jw. (13032)

24 or/18-23 (235698)

25 17 and 24 (0)

26 randomized controlled trial/ or controlled clinical trial/ (499925)

27 exp "clinical trial (topic)"/ (133263)

28 (randomi#ed or randomly or RCT$1 or placebo*).ti,ab,kw. (866049)

29 ((singl* or doubl* or trebl* or tripl*) adj (mask* or blind* or dumm*)).ti,ab,kw. (175856)

30 trial.ti. (179802)

31 or/26-30 (1227301)

32 17 and 31 (0)

33 (nonrandom* or non-random* or quasi-random* or quasi-experiment*).ti,ab,kw. (42762)

34 (nRCT or nRCTs or non-RCT$1).ti,ab,kw. (443)

35 (control* adj3 ("before and after" or "before after")).ti,ab,kw. (3623)

36 time series analysis/ (15115)

37 (time series adj3 interrupt*).ti,ab,kw. (1346)

38 controlled study/ (4536384)

39 (control* adj2 stud$3).ti,ab,kw. (207809)

40 control group/ (84744)

41 (control$ adj2 group$1).ti,ab,kw. (467804)

42 or/33-41 (4869615)

43 17 and 42 (0)

44 cohort analysis/ (191645)

45 cohort.ti,ab,kw. (414599)

46 retrospective study/ (383259)

47 longitudinal study/ (74311)

48 prospective study/ (278379)

49 (longitudinal or prospective or retrospective).ti,ab,kw. (1089493)

50 follow up/ (909974)

51 ((followup or follow-up) adj (study or studies)).ti,ab,kw. (55646)

52 population research/ (71971)

53 ((population or population-based) adj (study or studies or analys#s)).ti,ab,kw. (16836)

54 exp case control study/ (94573)

55 (case-control* or case-base or case-based or case-comparison or case-compeer or case-referent or case-referrent).ti,ab,kw. (109380)

56 (ecological adj (study or studies)).ti,ab,kw. (3359)

57 or/44-56 (2405618)

58 17 and 57 (0)

59 25 or 32 or 43 or 58 (0)

60 exp animal experimentation/ or exp models animal/ or exp animal experiment/ or nonhuman/ or exp vertebrate/ (21427592)

61 exp humans/ or exp human experimentation/ or exp human experiment/ (15736476)

62 60 not 61 (5692128)

63 59 not 62 (0)

64 (comment or editorial or interview).pt. (467286)

65 63 not 64 (0)

***************************

Cochrane Library

Search Name: Maternal Influenza - Incidence - DALYs

Date Run: 28/02/15 19:17:59.155

Description: WHO - 2015 Feb 28 - Final

ID Search Hits

#1 [mh "Influenza Human"] 1371

#2 (influenza* or flu or grippe):ti,ab,kw 5573

#3 [mh "Influenza A Virus"] 735

#4 [mh "Influenza B Virus"] 226

#5 (H1N1 or PH1N1 or H3N2 or AH1N1 or AH3N2):ti,ab,kw 824

#6 {or #1-#5} 5576

#7 [mh Pregnancy] 5855

#8 [mh "Pregnancy Complications"] 7906

#9 pregnan*:ti,ab,kw 27070

#10 (prenatal* or antenatal* or (ante next natal*) or antepartum or "ante partum" or perinatal* or (peri next natal*) or peripartum or "peri partum"):ti,ab,kw 6645

#11 maternal*:ti,ab,kw 8896

#12 {or #7-#11} 32109

#13 #6 and #12 102

#14 ("disability-adjusted" near/2 (year or years)):ti,ab,kw 36

#15 (DALY or DALYs):ti,ab,kw 38

#16 {or #14-#15} 52

#17 #13 and #16 0

CINAHL Plus with Full Text

| Search ID# | Search Terms | Actions |
| --- | --- | --- |
| S17 | (S14 OR S15) AND (S13 AND S16) | (0) |
| S16 | S14 OR S15 | (404) |
| S15 | TI ( DALY or DALYs ) OR AB ( DALY or DALYs ) | (308) |
| S14 | TI ( "disability-adjusted" W2 (year or years) ) OR AB ( "disability-adjusted" W2 (year or years) ) | (273) |
| S13 | S6 AND S12 | (832) |
| S12 | S7 OR S8 OR S9 OR S10 OR S11 | (163,307) |
| S11 | TI maternal* OR AB maternal* | (30,217) |
| S10 | TI ( prenatal* or antenatal* or (ante W1 natal*) or antepartum or "ante partum" or perinatal* or (peri W1 natal*) or peripartum or "peri partum" ) OR AB ( prenatal* or antenatal* or (ante W1 natal*) or antepartum or "ante partum" or perinatal* or (peri W1 natal*) or peripartum or "peri partum" ) | (27,155) |
| S9 | TI pregnan* OR AB pregnan* | (57,854) |
| S8 | MH "Pregnancy Complications+" | (51,616) |
| S7 | MH "Pregnancy+" | (128,977) |
| S6 | S1 OR S2 OR S3 OR S4 OR S5 | (17,577) |
| S5 | TI ( (H1N1 or PH1N1 or H3N2 or AH1N1 or AH3N2 ) OR AB ( (H1N1 or PH1N1 or H3N2 or AH1N1 or AH3N2 ) | (3,461) |
| S4 | MH "Influenza B Virus" | (184) |
| S3 | MH "Influenza A Virus+" | (3,834) |
| S2 | TI ( influenza* or flu or grippe ) OR AB ( influenza* or flu or grippe ) | (15,745) |
| S1 | MH "Influenza, Human+" | (5,287) |
