# Supplementary material for: Incidence of influenza virus infection among pregnant women: a systematic review
Source: BMC Pregnancy Childbirth. 2017 May 30;17:155. doi: 10.1186/s12884-017-1333-5 (PMC5450114; doi:10.1186/s12884-017-1333-5)
Supplement: Supplementary file 2 — Application of GRADE evaluation to various outcomes of systematic literature review of the incidence of influenza virus infection among pregnant women. (DOCX 151 kb) [file 12884_2017_1333_MOESM2_ESM.docx]

**Appendix B. Application of GRADE evaluation to various outcomes of systematic literature review of the incidence of influenza virus infection among pregnant women**

**Table 1. GRADE evaluation for serological studies describing Infection, including subclinical infection**

| **Profile of individual studies** | | **Comments** |
| --- | --- | --- |
| **Number of studies**  **Number of estimates** | 3  3 |  |
| **Estimated number of participants** | 3,925 | - For Grffiths study, only women with post-partum specimens were included |
| **Total number of infections** | 397 | - The testing methodology was different in each of the three studies |
| **Lowest point estimate (95% Confidence Intervals)**** | 483 (399-614) |  |
| **Highest point estimate (95% Confidence Intervals)**** | 1,097 (957-1,258) |  |
| **Newcastle Ottawa Score** |  |  |
| 0–5 | 0 |  |
| 6–7 | 3 |  |
| 8–9 (highest quality) | 0 |  |
| **Statistical heterogeneity across studies: *I^2^*= 95.57% (95% CI: 90.93-97.98)** | | |
| **GRADE assessment ^a,b^** | **Comments** | |
| **Phase of investigation** | Level 2 (high) | - All studies were observational studies |
| **GRADE criteria (based on narrative review, not meta-analysis)** |  | |
| **Study limitations:**   - **Downgrade by -1** if most evidence is from studies with moderate or unclear risk of bias for most bias domains (serious limitations). - **Downgrade by -2** if most evidence is from studies with high risk of bias for almost all bias domains (very serious limitations). | -1 | - Medium risk of bias because of the fact that women were sampled at different times of the year and follow-up serum was taken at different intervals and a different number of times in each of the 3 studies - **Downgrade by 1.** |
| **Inconsistency:** unexplained heterogeneity or variability in results across studies   - **Downgrade by -1** when estimates of the risk factor association with the outcome vary in direction (for example, some effects appear protective whereas others show risk) and the confidence intervals show no, or minimal overlap. | -1 | - See Forest plot. There is very high heterogeneity in results across studies, qualitatively and quantitatively (*I^2^*=96%). - The confidence intervals of the two studies only overlap. - **Downgrade by 1.** |
| **Indirectness:** the study sample, the prognostic factor, and/or the outcome in the primary studies do not accurately reflect the review question   - **Downgrade by -1** when: (1) the final sample only represents a subset of the population of interest; (2) when the complete breadth of the prognostic factor that is being considered in the review question is not well represented in the available studies; or (3) when the outcome that is being considered in the review question is not broadly represented. |  | - No change. |
| **Imprecision:**   - **Downgrade by -1** if the evidence is generated by a few studies involving a small number of participants and most of the studies provide imprecise results. - For narrative summary: Within-study imprecision: (1) sample size justification is not provided and there are less than 10 outcome events for each prognostic variable (for dichotomous outcomes), and (2) no precision in the estimation of the effect size within each primary study. Across study imprecision: there are few studies and small number of participants across studies. | -1 | - There are only 3 studies and a relatively small number of participants and cases; 2 studies are related to seasonal influenza and one addresses pandemic influenza. |
| **Publication bias:**   - **Downgrade by -1** unless the value of the risk/protective factor in predicting the outcome has been repetitively investigated, ideally by phase 2 and 3 studies. |  | - No change. |
| **Moderate/large effect size:**   - **Upgrade by +1** if moderate or large similar effect is reported by most studies. |  | - No change (most studies do not report large effect sizes and those that do have a high to very high risk of diagnostic ascertainment bias). |
| **Dose effect:**   - **Upgrade by +1** if possible gradient exists within and between primary studies. | N/A | - No change. |
| **GRADE: OVERALL QUALITY OF EVIDENCE**  **(+, very low; ++, low; +++, moderate; ++++, high)** | **+**  **Very low** |  |

^a^ Based on Huguet et al. [18] adaptation of GRADE evaluation framework.[19]

^b^ ✕ indicates serious limitations.

* per 10,000 pregnancies

**Table 2. GRADE evaluation for studies describing symptomatic Infection**

| **Profile of individual studies** | | **Comments** |
| --- | --- | --- |
| **Number of studies**  **Number of estimates** | 3  3 |  |
| **Estimated number of participants** | 297,086 | - Because the Jamieson study was included, only the numerator was included |
| **Total number of infections** | 282 |  |
| **Lowest point estimate (95% Confidence Intervals)**** | 0.10 (0.07-0.14) |  |
| **Highest point estimate (95% Confidence Intervals)**** | 486 (375-630) |  |
| **Newcastle Ottawa Score** |  |  |
| 0–5 | 0 |  |
| 6–7 | 0 |  |
| 8–9 (highest quality) | 1 |  |
| **JBI Assessment score** | 7/8 |  |
| Cochrane Collaboration tool for assessing risk of bias in randomised trials | Low Risk of Bias |  |
| **Statistical heterogeneity across studies: *I^2^*= 99.74% (95% CI 99.69-99.77)** | | |
| **GRADE assessment ^a,b^** | **Comments** | |
| **Phase of investigation (** | Moderate | - Varied study types |
| **GRADE criteria (based on narrative review, not meta-analysis)** |  | |
| **Study limitations:**   - **Downgrade by -1** if most evidence is from studies with moderate or unclear risk of bias for most bias domains (serious limitations). - **Downgrade by -2** if most evidence is from studies with high risk of bias for almost all bias domains (very serious limitations). | -1 | - Two of the three studies had major risk of diagnostic ascertainment bias - **Downgrade by 1.** |
| **Inconsistency:** unexplained heterogeneity or variability in results across studies   - **Downgrade by -1** when estimates of the risk factor association with the outcome vary in direction (for example, some effects appear protective whereas others show risk) and the confidence intervals show no, or minimal overlap. | -1 | - There is very high heterogeneity in results across studies, qualitatively and quantitatively (*I^2^*=99%). - None of the confidence intervals overlap - **Downgrade by 1.** |
| **Indirectness:** the study sample, the prognostic factor, and/or the outcome in the primary studies do not accurately reflect the review question   - **Downgrade by -1** when: (1) the final sample only represents a subset of the population of interest; (2) when the complete breadth of the prognostic factor that is being considered in the review question is not well represented in the available studies; or (3) when the outcome that is being considered in the review question is not broadly represented. |  | - No change. |
| **Imprecision:**   - **Downgrade by -1** if the evidence is generated by a few studies involving a small number of participants and most of the studies provide imprecise results. - For narrative summary: Within-study imprecision: (1) sample size justification is not provided and there are less than 10 outcome events for each prognostic variable (for dichotomous outcomes), and (2) no precision in the estimation of the effect size within each primary study. Across study imprecision: there are few studies and small number of participants across studies. | -1 | - There are only 3 studies and a relatively small number of participants and cases; 2 studies are related to seasonal influenza and one addresses pandemic influenza. |
| **Publication bias:**   - **Downgrade by -1** unless the value of the risk/protective factor in predicting the outcome has been repetitively investigated, ideally by phase 2 and 3 studies. |  | - No change. |
| **Moderate/large effect size:**   - **Upgrade by +1** if moderate or large similar effect is reported by most studies. |  | - No change (most studies do not report large effect sizes and those that do have a high to very high risk of diagnostic ascertainment bias). |
| **Dose effect:**   - **Upgrade by +1** if possible gradient exists within and between primary studies. | N/A | - No change. |
| **GRADE: OVERALL QUALITY OF EVIDENCE**  **(+, very low; ++, low; +++, moderate; ++++, high)** | **+**  **Very low** |  |

^a^ Based on Huguet et al. [18] adaptation of GRADE evaluation framework.[19]

^b^ indicates serious limitations.

* per 10,000 maternities

**Table 3. GRADE evaluation for studies describing influenza-associated hospital admission**

| **Profile of individual studies** | | **Comments** |
| --- | --- | --- |
| **Number of studies**  **Number of estimates** | 4  4 |  |
| **Estimated number of participants** | 610,153 | - Because Jamieson and Creanga studies were case series, only the numerators were included |
| **Total number of infections** | 488 |  |
| **Lowest point estimate (95% Confidence Intervals)**** | 0.04 (0.02-0.07) |  |
| **Highest point estimate (95% Confidence Intervals)**** | 7.7 (6.7-8.7) |  |
| **Newcastle Ottawa Score** |  |  |
| 0–5 | 0 |  |
| 6–7 | 3 |  |
| 8–9 (highest quality) | 2 |  |
| **JBI Assessment score** | 6/7 (2) |  |
| **Statistical heterogeneity across studies: *I^2^*= 99.77% (95% CI 99.71-99.82%)**** | | |
| **GRADE assessment ^a,b^** | **Comments** | |
| **Phase of investigation** | Level 2 (high) | - All studies were observational studies |
| **GRADE criteria (based on narrative review, not meta-analysis)** |  | |
| **Study limitations:**   - **Downgrade by -1** if most evidence is from studies with moderate or unclear risk of bias for most bias domains (serious limitations). - **Downgrade by -2** if most evidence is from studies with high risk of bias for almost all bias domains (very serious limitations). | -2 | - Significant risk of diagnostic ascertainment bias related to likely frequency of testing patients and variability of reporting results to city, state and federal public health authorities. In addition, reporting period was variable in the four studies. - **Downgrade by -2** |
| **Inconsistency:** unexplained heterogeneity or variability in results across studies   - **Downgrade by -1** when estimates of the risk factor association with the outcome vary in direction (for example, some effects appear protective whereas others show risk) and the confidence intervals show no, or minimal overlap. | -1 | - There is very high heterogeneity in results across studies, qualitatively and quantitatively (*I^2^*>99%). Methodology was different in all studies, and not all confidence levels overlap. - **Downgrade by 1.** |
| **Indirectness:** the study sample, the prognostic factor, and/or the outcome in the primary studies do not accurately reflect the review question   - **Downgrade by -1** when: (1) the final sample only represents a subset of the population of interest; (2) when the complete breadth of the prognostic factor that is being considered in the review question is not well represented in the available studies; or (3) when the outcome that is being considered in the review question is not broadly represented. |  | - No change. |
| **Imprecision:**   - **Downgrade by -1** if the evidence is generated by a few studies involving a small number of participants and most of the studies provide imprecise results. - For narrative summary: Within-study imprecision: (1) sample size justification is not provided and there are less than 10 outcome events for each prognostic variable (for dichotomous outcomes), and (2) no precision in the estimation of the effect size within each primary study. Across study imprecision: there are few studies and small number of participants across studies. |  | - There are only 4 studies, but all studies describe burden related to 2009 pandemic influenza. No change. |
| **Publication bias:**   - **Downgrade by -1** unless the value of the risk/protective factor in predicting the outcome has been repetitively investigated, ideally by phase 2 and 3 studies. |  | - No change. |
| **Moderate/large effect size:**   - **Upgrade by +1** if moderate or large similar effect is reported by most studies. | N/A |  |
| **Dose effect:**   - **Upgrade by +1** if possible gradient exists within and between primary studies. | N/A | - No change. |
| **GRADE: OVERALL QUALITY OF EVIDENCE**  **(+, very low; ++, low; +++, moderate; ++++, high)** | **+**  **Very low** |  |

^a^ Based on Huguet et al. [18] adaptation of GRADE evaluation framework.[19]

^b^ ✕ indicates serious limitations.

* per 10,000 maternities

**CIs calculated using Test method

**Table 4. GRADE evaluation for studies reporting influenza-associated ICU admission**

| **Profile of individual studies** | | **Comments** |
| --- | --- | --- |
| **Number of studies**  **Number of estimates** | 4  4 |  |
| **Estimated number of participants** | 296,069 | - Because the Jamieson, Creanga and Knight studies were case series, only the numerators were included |
| **Total number of infections** | 137 |  |
| **Lowest point estimate (95% Confidence Intervals)**** | 0.01(0.00-0.03) |  |
| **Highest point estimate (95% Confidence Intervals)**** | 6.8 (5.2-8.8) |  |
| **Newcastle Ottawa Score** |  |  |
| 0–5 | 0 |  |
| 6–7 | 0 |  |
| 8–9 (highest quality) | 1 |  |
| **JBI Assessment score** | 6/7 (3) |  |
| **Statistical heterogeneity across studies: *I^2^*= 99.00% (95% CI 98.77-99.17%)** | | |
| **GRADE assessment ^a,b^** | **Comments** | |
| **Phase of investigation** | Low | - Most studies were case studies; only one retrospective observational study |
| **GRADE criteria (based on narrative review, not meta-analysis)** |  | |
| **Study limitations:**   - **Downgrade by -1** if most evidence is from studies with moderate or unclear risk of bias for most bias domains (serious limitations). - **Downgrade by -2** if most evidence is from studies with high risk of bias for almost all bias domains (very serious limitations). | -2 | - Significant risk of diagnostic ascertainment bias related to likely frequency of testing patients and variability of reporting results to city, state and federal public health authorities. In addition, reporting period was variable in the four studies. - **Downgrade by 2** |
| **Inconsistency:** unexplained heterogeneity or variability in results across studies   - **Downgrade by -1** when estimates of the risk factor association with the outcome vary in direction (for example, some effects appear protective whereas others show risk) and the confidence intervals show no, or minimal overlap. |  | - No change |
| **Indirectness:** the study sample, the prognostic factor, and/or the outcome in the primary studies do not accurately reflect the review question   - **Downgrade by -1** when: (1) the final sample only represents a subset of the population of interest; (2) when the complete breadth of the prognostic factor that is being considered in the review question is not well represented in the available studies; or (3) when the outcome that is being considered in the review question is not broadly represented. |  | - No change. |
| **Imprecision:**   - **Downgrade by -1** if the evidence is generated by a few studies involving a small number of participants and most of the studies provide imprecise results. - For narrative summary: Within-study imprecision: (1) sample size justification is not provided and there are less than 10 outcome events for each prognostic variable (for dichotomous outcomes), and (2) no precision in the estimation of the effect size within each primary study. Across study imprecision: there are few studies and small number of participants across studies. |  | - There are only 4 studies, but three of the four studies have similar findings with overlapping confidence intervals. All studies address pandemic influenza; no studies address seasonal influenza. No change. |
| **Publication bias:**   - **Downgrade by -1** unless the value of the risk/protective factor in predicting the outcome has been repetitively investigated, ideally by phase 2 and 3 studies. |  | - No change. |
| **Moderate/large effect size:**   - **Upgrade by +1** if moderate or large similar effect is reported by most studies. | N/A |  |
| **Dose effect:**   - **Upgrade by +1** if possible gradient exists within and between primary studies. | N/A |  |
| **GRADE: OVERALL QUALITY OF EVIDENCE**  **(+, very low; ++, low; +++, moderate; ++++, high)** | **+**  **Very low** |  |

^a^ Based on Huguet et al. [18] adaptation of GRADE evaluation framework.[19]

^b^ ✕ indicates serious limitations.

* per 10,000 maternities

**Table 5. GRADE evaluation of studies reporting influenza-associated death**

| **Profile of individual studies** | | **Comments** |
| --- | --- | --- |
| **Number of studies**  **Number of estimates** | 4  4 |  |
| **Estimated number of participants** | 295,954 | - Because the Jamieson, Creanga and Knight studies were case series, only the numerators were included |
| **Total number of infections** | 21 |  |
| **Lowest point estimate (95% Confidence Intervals)**** | 0.003 (0.000-0.021) |  |
| **Highest point estimate (95% Confidence Intervals)**** | 0.69 (0.26-1.51) |  |
| **Newcastle Ottawa Score** |  |  |
| 0–5 | 0 |  |
| 6–7 | 0 |  |
| 8–9 (highest quality) | 1 |  |
| **JBI Assessment score** | 6/7 (3) |  |
| **Statistical heterogeneity across studies: *I^2^*= 93.11% (95% CI: 87.37-95.56)** | | |
| **GRADE assessment ^a,b^** | **Comments** | |
| **Phase of investigation** | Low | - Most studies were case studies; only one retrospective observational study |
| **GRADE criteria (based on narrative review, not meta-analysis)** |  | |
| **Study limitations:**   - **Downgrade by -1** if most evidence is from studies with moderate or unclear risk of bias for most bias domains (serious limitations). - **Downgrade by -2** if most evidence is from studies with high risk of bias for almost all bias domains (very serious limitations). | -2 | - Significant risk of diagnostic ascertainment bias related to likely frequency of testing patients and variability of reporting results to city, state and federal public health authorities. In addition, reporting period was variable in the four studies. - **Downgrade by 2.** |
| **Inconsistency:** unexplained heterogeneity or variability in results across studies   - **Downgrade by -1** when estimates of the risk factor association with the outcome vary in direction (for example, some effects appear protective whereas others show risk) and the confidence intervals show no, or minimal overlap. | 0 | - There is high heterogeneity in results across studies, qualitatively and quantitatively (*I^2^*=99%). - The confidence intervals of three of the four studies overlap. - **No change** |
| **Indirectness:** the study sample, the prognostic factor, and/or the outcome in the primary studies do not accurately reflect the review question   - **Downgrade by -1** when: (1) the final sample only represents a subset of the population of interest; (2) when the complete breadth of the prognostic factor that is being considered in the review question is not well represented in the available studies; or (3) when the outcome that is being considered in the review question is not broadly represented. |  | - No change. |
| **Imprecision:**   - **Downgrade by -1** if the evidence is generated by a few studies involving a small number of participants and most of the studies provide imprecise results. - For narrative summary: Within-study imprecision: (1) sample size justification is not provided and there are less than 10 outcome events for each prognostic variable (for dichotomous outcomes), and (2) no precision in the estimation of the effect size within each primary study. Across study imprecision: there are few studies and small number of participants across studies. | -1 | - There are only 4 studies and the sample size for the 3 case series include < 10 subjects. |
| **Publication bias:**   - **Downgrade by -1** unless the value of the risk/protective factor in predicting the outcome has been repetitively investigated, ideally by phase 2 and 3 studies. |  | - No change. |
| **Moderate/large effect size:**   - **Upgrade by +1** if moderate or large similar effect is reported by most studies. | N/A |  |
| **Dose effect:**   - **Upgrade by +1** if possible gradient exists within and between primary studies. | N/A |  |
| **GRADE: OVERALL QUALITY OF EVIDENCE**  **(+, very low; ++, low; +++, moderate; ++++, high)** | **+**  **Very low** |  |

^a^ Based on Huguet et al. [18] adaptation of GRADE evaluation framework.[19]

^b^ ✕ indicates serious limitations.

* per 10,000 maternities
